# Identification of lipid-phosphatidylserine (PS) as the target of unbiasedly selected cancer specific peptide-peptoid hybrid PPS1

**DOI:** 10.18632/oncotarget.8929

**Published:** 2016-04-22

**Authors:** Tanvi J. Desai, Jason E. Toombs, John D. Minna, Rolf A. Brekken, Damith Gomika Udugamasooriya

**Affiliations:** ^1^ Department of Pharmacological and Pharmaceutical Sciences, University of Houston, Houston, TX 77204, USA; ^2^ Department of Cancer Systems Imaging, MD Anderson Cancer Center, Houston, TX 77030, USA; ^3^ Hamon Center for Therapeutic Oncology Research, University of Texas Southwestern Medical Center, Dallas, TX 75390, USA; ^4^ Simmons Comprehensive Cancer Center, University of Texas Southwestern Medical Center, Dallas, TX 75390, USA; ^5^ Department of Pharmacology, University of Texas Southwestern Medical Center, Dallas, TX 75390, USA; ^6^ Department of Internal Medicine, University of Texas Southwestern Medical Center, Dallas, TX 75390, USA; ^7^ Department of Surgery, University of Texas Southwestern Medical Center, Dallas, TX 75390, USA

**Keywords:** phosphatidylserine, peptoids, non-protein biomarkers, anti-cancer agents, lung cancer

## Abstract

Phosphatidylserine (PS) is an anionic phospholipid maintained on the inner-leaflet of the cell membrane and is externalized in malignant cells. We previously launched a careful unbiased selection targeting biomolecules (e.g. protein, lipid or carbohydrate) distinct to cancer cells by exploiting HCC4017 lung cancer and HBEC30KT normal epithelial cells derived from the same patient, identifying HCC4017 specific peptide-peptoid hybrid PPS1. In this current study, we identified PS as the target of PPS1. We validated direct PPS1 binding to PS using ELISA-like assays, lipid dot blot and liposome based binding assays. In addition, PPS1 recognized other negatively charged and cancer specific lipids such as phosphatidic acid, phosphatidylinositol and phosphatidylglycerol. PPS1 did not bind to neutral lipids such as phosphatidylethanolamine found in cancer and phosphatidylcholine and sphingomyelin found in normal cells. Further we found that the dimeric version of PPS1 (PPS1D1) displayed strong cytotoxicity towards lung cancer cell lines that externalize PS, but not normal cells. PPS1D1 showed potent single agent anti-tumor activity and enhanced the efficacy of docetaxel in mice bearing H460 lung cancer xenografts. Since PS and anionic phospholipid externalization is common across many cancer types, PPS1 may be an alternative to overcome limitations of protein targeted agents.

## INTRODUCTION

**Figure 1:**
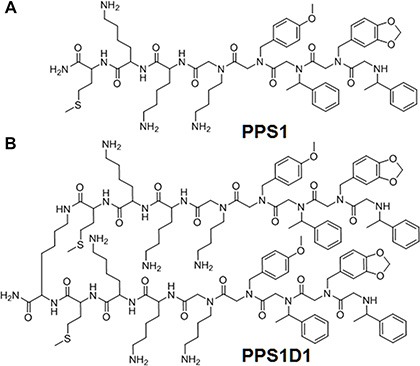
Chemical structures of PPS1 and PPS1D1 (**A**) PPS1 monomer and (**B**) PPS1D1, a dimer containing two PPS1 molecules linked with Lysine residue.

Conventional drug development targeting cell surface proteins is challenging in oncology due to the diversity and complexity of cancer [1–3]. The heterogeneity of protein expression and cross-talk/compensation between signaling cascades present significant hurdles for the development of therapeutic agents that provide durable efficacy and are broadly effective [4]. We hypothesized that identifying compounds that target non-protein based cell surface bio-molecules that are widely expressed across many cancer types would address some of these challenges. Therefore, we performed a unique unbiased selection approach to target biomolecules such as proteins, lipids or carbohydrates present on the cancer cell surface, but not found or less abundant on normal cells. We utilized our on-bead two-color (OBTC) combinatorial cell screen to select peptide-peptoid hybrids that discriminate cell surface targets in closely related cell populations [5]. This screening strategy is unbiased in terms of the nature of target selection allowing equal chance to recognize a protein, lipid or a carbohydrate specific to cancer cells. The OBTC cell screen was performed using a lung cancer cell line (HCC4017) and normal bronchial epithelial cells (HBEC30KT) derived from the same patient. A library of ~400,000 peptide-peptoid hybrids was screened against a mixture of HCC4017 and HBEC30KT cells. The cells were pre-labeled with fluorescent quantum dots such that HCC4017 cells were red and HBEC30KT cells were green. Beads that only bound to red stained HCC4017 cells were selected for further characterization. We identified a peptide-peptoid hybrid called PPS1 (Figure 1A) that binds HCC4017 lung cancer cells with limited or no binding to normal HBEC30KT cells. The dimeric version of PPS1, PPS1D1 (Figure 1B) displayed strong cytotoxic activity on HCC4017 cells, but not on HBEC30KT cells. Furthermore, PPS1D1 strongly accumulated in HCC4017 lung cancer xenografts [5].

Target identification of an unbiasedly selected compound is challenging and the first assumption is that the compound binds to a specific protein. A typical approach to identify the targeted protein is mass spectrometry after compound facilitated precipitation [6–9]. We attempted this path without success (data not shown). However, we evaluated targets of other molecular classes such as lipids and carbohydrates that may be found in cancer. In particular, anionic phospholipids [10, 11], sialic acid residues [12] and heparin sulfates [13, 14] are some known examples of other molecular classes that are elevated on the cancer cell surface.

Herein we describe the target identification and binding of PPS1 to anionic phospholipids, principally, phosphatidylserine (PS). Typically the plasma membrane consists of phosphatidylcholine (PC) and sphingomyelin (SM) in the outer leaflet, while PS and phosphatidylethanolamine (PE) are segregated to the inner leaflet [15, 16]. This distribution is actively maintained but dynamically changes in response to physiological and pathophysiological events. Movement of lipid across the membrane is controlled by aminophospholipid translocases, scramblases, ATP binding cassette group of transporters and Ca2^+^ concentration [17, 18], and is well studied during apoptosis, malignancy and cell damage [19–22]. The anionic phospholipid PS has been reported as a marker of tumor vasculature [10, 11, 23, 24]. This is because PS is flipped to the outer leaflet of the plasma membrane in endothelial cells in the tumor microenvironment and also in some cancer cells due to oxidative stress, hypoxia/re-oxygenation, cytokine activation, cell trafficking and tumor cell metabolites. PS is also externalized during apoptosis, necrosis, and cell activation [10, 23, 25]. Depending on tumor type, up to 50% of the blood vessels in the tumor can externalize PS [10, 23, 24, 26, 27]. Further, PS exposure on tumor vasculature is elevated after therapy with chemotherapy, radiation, androgen deprivation or small molecules [27, 28].

There are only a small number of PS targeted peptides, antibodies and small molecules that have been reported to date. The most widely studied PS-binding probe is annexin V, a 35.8 kDa protein that binds PS in a calcium-dependent manner with nanomolar affinity [29, 30]. A PS binding peptide identified by screening M13 phage library was used for H460 tumors imaging in mice [31]. Another peptide was identified by screening a library of compounds for their binding to PS-coated surface plasmon resonance sensor chips [32]. This peptide was conjugated with 99 mTc and was shown to bind to cancer cells. Zinc containing small molecules targeting PS has also been used for optical imaging of tumors [33, 34]. Bavituximab, a chimeric monoclonal antibody that binds PS via a co-factor, β2-glycoprotein-1 has been used for the therapy and imaging of solid tumors in preclinical models and is currently under clinical testing in cancer patients [23, 24, 35].

While small molecules, peptides and antibodies are also display their own weaknesses in terms of developing as probes and/or drugs, we were interested in exploring emerging molecular class of peptidomimetics called peptoids. Peptoids have peptide-like characteristics and have emerged as important alternative molecules for anti-cancer drug-lead development. Peptoids have peptide like backbone but each residue is N-substituted glycine, which is equivalent to an amino acid of a peptide [36–39]. The R group of a peptoid residue is placed on nitrogen instead of the alpha carbon in a peptide. This arrangement confers protease resistance, cell permeability and reduced immunogenicity [40, 41]. Large peptoid libraries containing millions of molecules can be rapidly and easily synthesized at low cost [42–44]. Peptoids have been reported as potential drug leads targeting various cancer targets [37–39, 43] and imaging agent carries [45]. In this study we describe the target identification of our peptide-peptoid hybrid PPS1 and demonstrate that PPS1 has potential as an anti-cancer therapeutic.

## RESULTS AND DISCUSSION

### PS exposure on HCC4017 lung cancer cells

**Figure 2:**
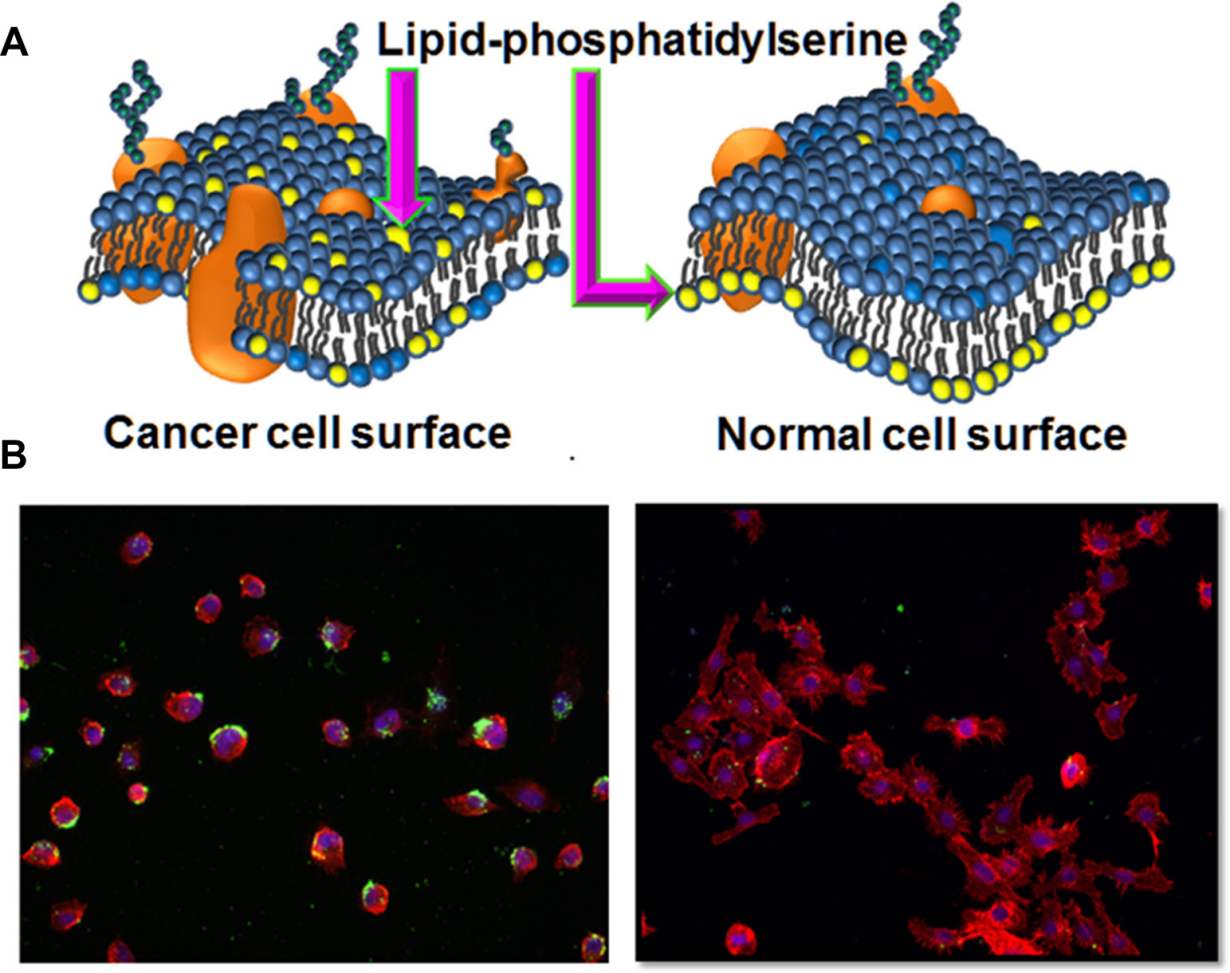
Lipid-PS expression difference between cancer vs normal cells (**A**) Schematic representation of membrane lipid asymmetry in cancer and normal cells and lipid-PS externalization on cancer cells. (**B**) Staining of HCC4017 (left) and HBEC30KT (right) with PS targeting bavituximab antibody. Only HCC4017 cells express PS.

The amphipathic nature of PPS1 (Figure 1A and Supplementary Figure 1) suggested that this compound may target cell membrane lipids, as many anti-microbial peptides reported to date typically display the same structural features [46–48]. As described above, PS is well-known to be externalized in the tumor vascular endothelial cells and on some tumor cells as depicted in Figure 2A compared to normal cells. In our previous study [5], PPS1 was selected for binding to HCC4017 cells over HBEC30KT cells, thus we first evaluated the level of PS exposure in HCC4017 and HBEC30KT cells using immunocytochemistry with the PS targeting antibody bavituximab. HCC4017 cells were robustly positive for bavituximab staining while HBEC30KT showed little to no staining with this PS targeting agent (Figure 2B), indicating significantly elevated levels of PS on outer leaflet of HCC4017 compared to normal HBEC30KT. This observation is consistent with PS exposure on other cancer cell types such as lymphoma, melanoma and colon carcinoma cell lines [49, 50].

### PPS1D1 binds PS

**Figure 3:**
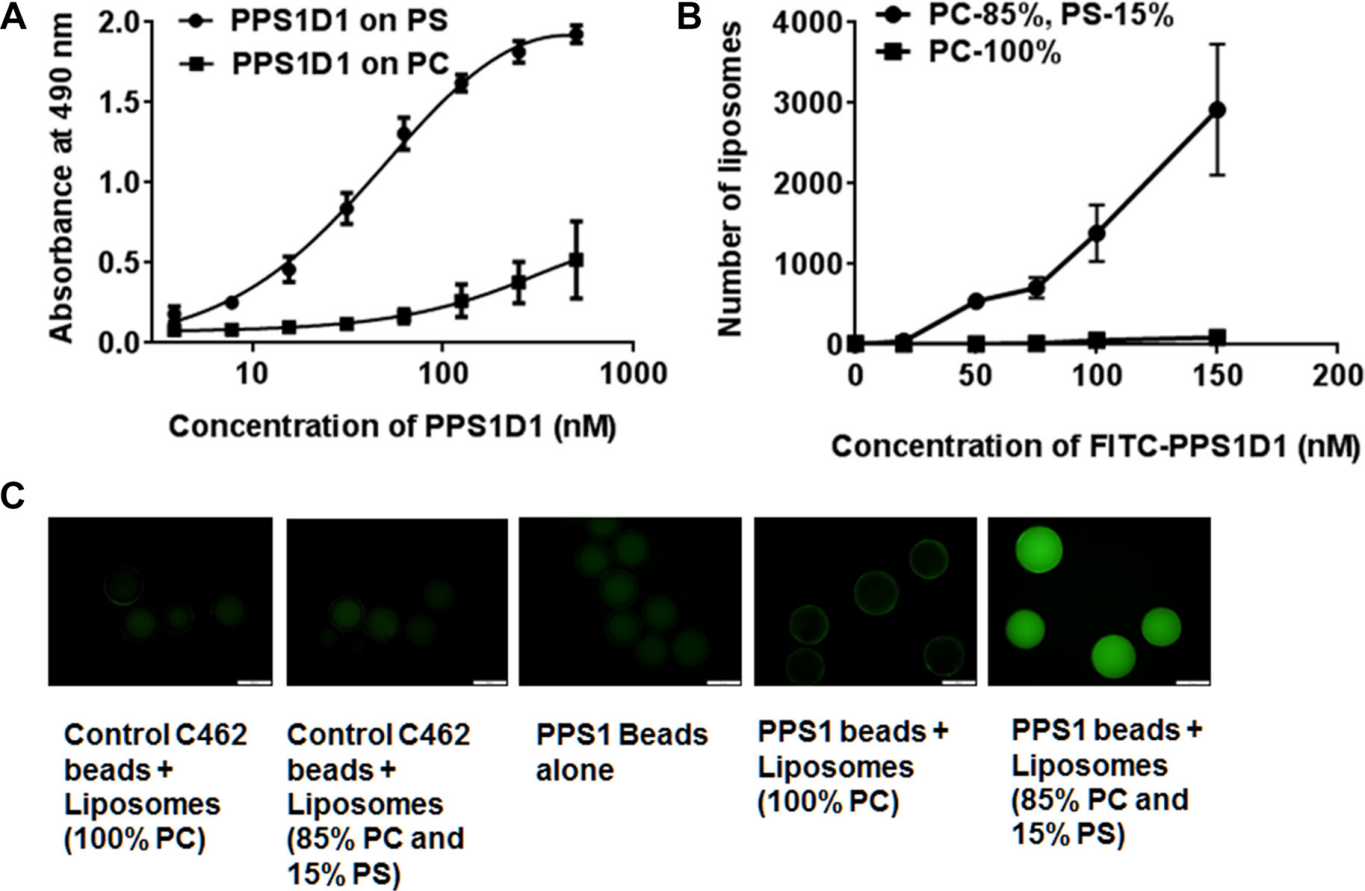
PPS1D1 recognize lipid-PS (**A**) ELISA binding assay of FITC-PPS1D1 with phosphatidylcholine (PC) and phosphatidylserine (PS) indicates that FITC-PPS1D1 only binds to PS. (**B**) Binding of liposomes made of 100% PC and 85% PC–15% PS to PPS1D1-FITC. Only 15% PS containing liposomes bound to FITC-PPS1D1 (Error bars represent standard deviation). (**C**) Binding of liposomes made of 100% PC and 85% PC–15% PS to PPS1 and control PC462 carrying tentagel beads. Only 15% PS containing liposomes bound to PPS1 beads, but not liposomes with no PS (100% PC). Control PC462 does not bind to both liposome types.

Since PS is selectively exposed on the surface of HCC4017 cells compared with HBEC30KT, we examined if PPS1D1 (Figure 1B and Supplementary Figure 2) binds to PS directly. In an ELISA-like assay, PS and PC were coated separately on 96-well plates, biotinylated PPS1D1 (Supplementary Figure 3) was introduced in a concentration gradient and the bound compound was detected using standard streptavidin-HRP system. We observed that PPS1D1 bound to PS at K_D_ ~ 55 nM with very high specificity over the PC in a concentration-dependent manner (Figure 3A).

While an ELISA-like assay using purified components provides some information regarding binding characteristics, we predict that the cellular arrangement and dynamics of PS and PC might be different in a lipid bilayer. To partially address this issue, we extended our binding studies to liposomes with varying concentrations of PS. We created liposomes with 100% PC and 85% PC – 15% PS. These liposomes were incubated with fluorescein labeled PPS1D1 (FITC-PPS1D1, Supplementary Figure 4) at 20, 50, 75, 100 and 150 nM for 1 hour and the fluorescein signal was detected by flow cytometry. FITC-PPS1D1 specifically bound to liposomes that contained 15% PS but did not bind liposomes that were 100% PC (Figure 3B). These results demonstrated that PPS1D1 binds PS over PC. To confirm these observations, we synthesized PPS1 and non-PS-binding control compound PC462 [5] (Supplementary Figures 5, 7 and 8) on Tentagel beads and exposed those beads to liposomes with 100% PC and 85% PC – 15% PS that were incorporated with fluorophore 7-nitro-2-1,3-benzoxadiazol-4-yl (NBD) dye. As shown in Figure 3C, only the beads with PPS1 exposed to liposomes with 85% PC – 15% PS lit up indicating PPS1 binds to PS. No binding was observed on the beads with PPS1 exposed to liposomes with 100% PC or beads with control compounds (Figure 3C).

We conducted multiple competition assays to determine whether PPS1 (or PPS1D1) competes with Annexin V, a known PS binding agent. In ELISA-like competitive binding assay (Supplementary Figure 9) Annexin V did not compete with PPS1D1 for binding to PS. We also evaluated competition between Annexin V and PPS1 for binding to PS in liposomes using flow cytometry. Again in these assays there was no competition between Annexin V and PPS1 for binding to PS (Supplementary Figures 10 and 11). Finally, we demonstrated that Annexin V did not reduce the binding of NBD –PS containing liposomes on PPS1 displaying tentagel beads (Supplementary Figure 12). Prior reports have also found that PS targeting antibodies, including the IgM 9D2, do not cross block the binding of Annexin V to PS [10]. The lack of competition between PPS1 or antibody PS targeting agents with Annexin V for PS binding may be due to the different binding modes of these agents. Annexin V requires calcium to bind to PS. PS targeting agents such as 9D2 bind PS via the bridging protein β2-glycoprotein-1. However, PPS1 directly binds PS. Also, PS-ligand binding may be much more complex than that of a typical protein-ligand binding. Typical protein-ligand binding occurs via a defined binding pocket, which facilitates clear competition by other ligands that interact with the same binding pocket. But PS is a lipid in a fluid membrane, which has no defined macromolecular structure further complicating possible binding modes.

We next compared the structural features of PPS1 and PS. PPS1 monomer has three positively charged residues aligned together and a hydrophobic region with four consecutive aromatic rings (Figure 1A), while the PPS1D1 dimer has twice the amount of those positive and hydrophobic regions. This structure is likely to form electrostatic and hydrophobic interactions with opposing negatively charged head groups on PS and its hydrophobic tail region, suggesting that PPS1 and PPS1D1 interact with negatively charged phospholipids. To test this, we replaced one of the positively charged lysine residues (3rd residue from C-terminal) with glutamate (which will bring opposing negative charge) and observed a major reduction of the binding activity as shown in Supplementary Figure 13.

### PPS1D1 recognizes negatively charged phospholipids

**Figure 4:**
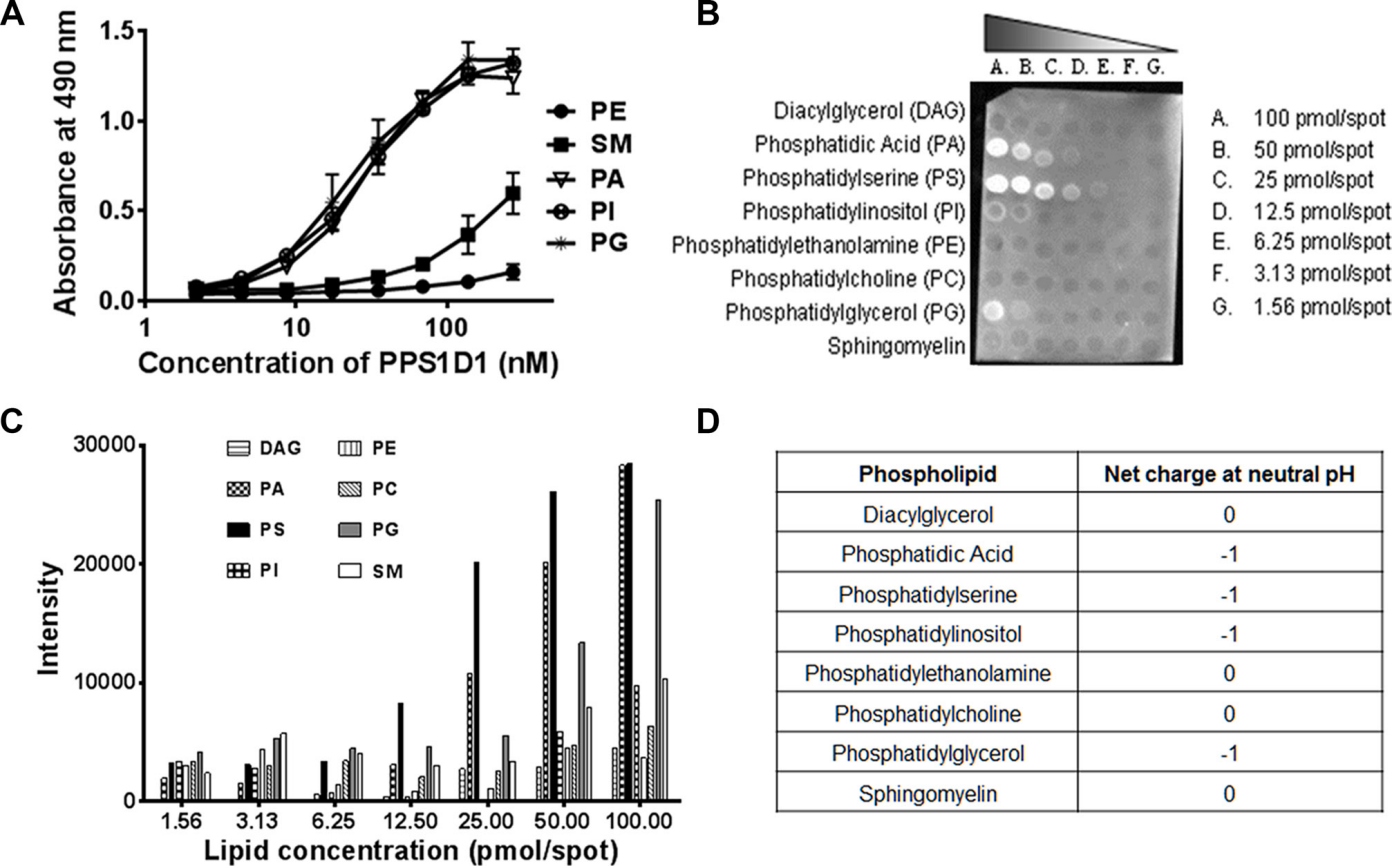
PPS1D1 binding studies on large panel of lipids (**A**) ELISA binding assay of PPS1D1-FITC with Phosphatidylethanolamine (PE), Sphingomyelin (SM), Phosphatidic Acid (PA), Phosphatidylinositol (PI) and Phosphatidylglycerol (PG). Only PA, PI and PG showed binding to PPS1D1-FITC (Error bars represent standard deviation) (**B**) Lipid dot blot showing binding of biotinylated-PPS1D1 with membrane phospholipids PS, PA, PG and PI, but not to PC, DAG, PE and SM. (**C**) Quantification of lipid-blot assay figure shown in (B). (**D**) Net charges of PA, PE, PC, PS, PG, PI and DAG lipids at neutral pH *(Adapted from, Lehninger Principles of Biochemistry, 5th Edition. Chapter 10, pg:351).*

One of the major questions arising at this point is how PPS1 or PPS1D1 specifically recognizes PS over PC, as both lipids have negatively charged phosphate head groups. To address this specificity concern we expanded our ELISA-like binding assay to include other membrane phospholipids such as phosphatidylethanolamine (PE), sphingomyelin (SM), phosphatidic acid (PA), phosphatidylinositol (PI) and phosphatidylglycerol (PG). As shown in Figure 4A, PPS1 displayed binding to PA, PI and PG but did not bind to PE and SM. Interestingly, all of the lipids bound by PPS1 (PS, PA, PI and PG) have an overall negative charge as compared to unbound PC, PE and SM that are neutral at physiological pH (Table shown in Figure 4D). We propose that this additional negative charge is responsible for the interaction with the positively charged region of PPS1 or PPS1D1, while the hydrophobic regions of PPS1 or PPS1D1 may interact with hydrophobic tail groups of the lipids through van der Waals forces. To validate these results on a different platform, we investigated PPS1D1 binding to phospholipids at different lipid concentrations using commercially available membrane lipid arrays, lipid dot blots (Echelon, USA). The membrane was treated with biotin labeled PPS1D1 and binding was detected by immunoblotting with streptavidin-HRP. PPS1D1 showed the strongest binding to PS, while PA, PG and PI followed with weaker binding (Figure 4B and 4C). This assay confirmed that PPS1D1 does not bind to PC, PE, SM or diacylglycerol (DAG) and recapitulated the ELISA-like assay. Furthermore, the binding characteristics of PPS1D1 are varied on PS, PA, PG and PI in the lipid blot assay and this suggests that PPS1D1 may have a secondary structure beyond a simple linear sequence. It has been reported that PS can form a bilayer lamellar and reverse hexagonal phase [51]. PS is mainly in a lamellar phase after hydration at physiological pH [52]. By validating our binding through an ELISA-like assay and liposomes, we have given an equal opportunity to PPS1D1 to bind to each phase of PS.

### Activity validation of PPS1D1 on series of lung cancer cell lines

**Figure 5:**
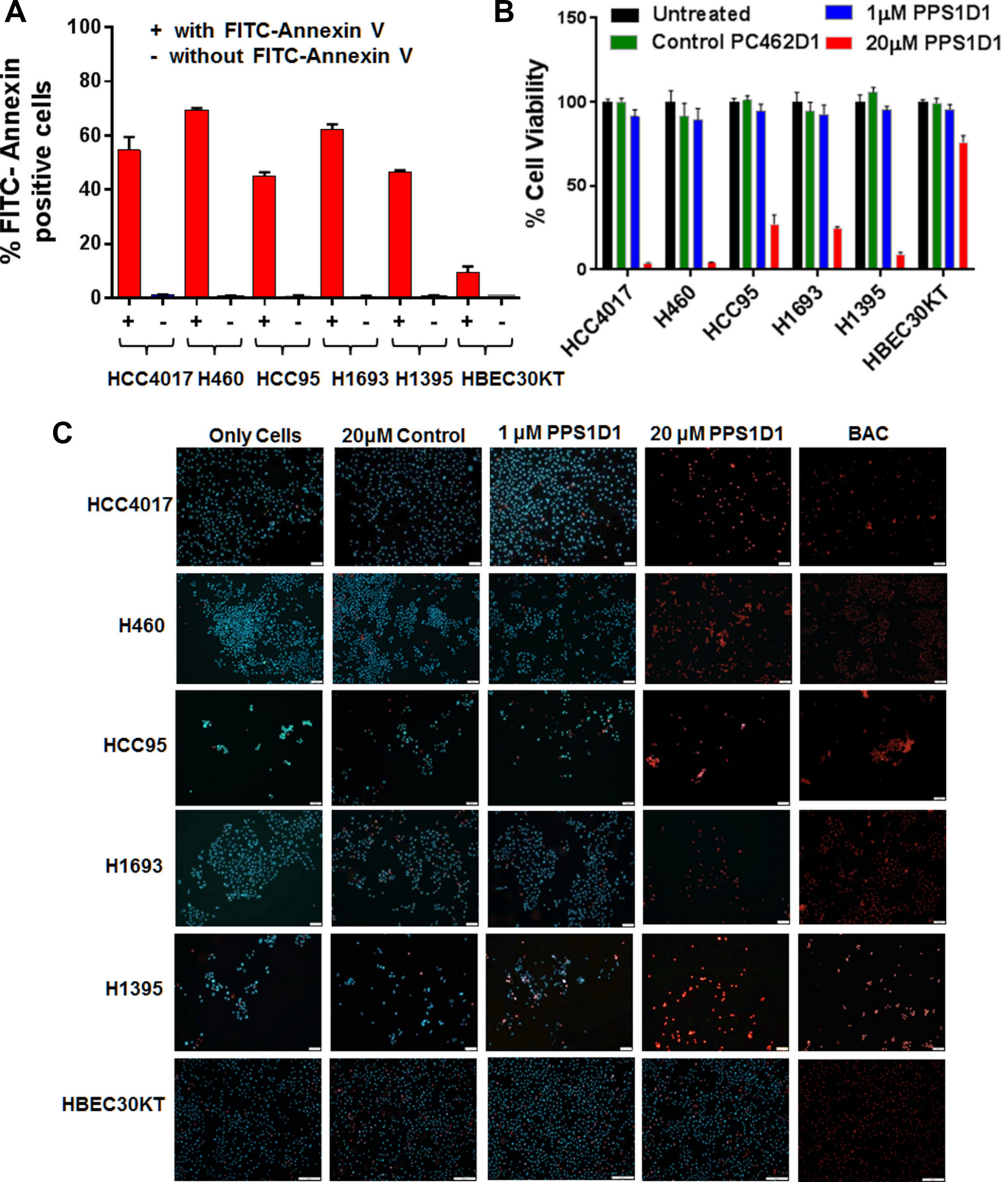
PPS1D1 binding and activity evaluation on panel of lung cancer cells (**A**) PS expression levels of lung cancer cell lines HCC4017, H460, HCC95, H1693, H1395 and normal HBEC30KT by binding with FITC-Annexin V. Lung cancer cells exhibited high PS levels while HBEC30KT has lower levels of PS (Error bars represent standard deviation). (**B**) Standard MTS cell viability data for the treatment of PPS1D1 and control PC462D1 on same lung cancer cells lines and HBEC30KT cells shown in (A). PPS1D1 at 20 μM caused strong cell cytotoxicity on cancer cells, but not on HBEC30KT. (**C**) Treatment of same lung cancer cells lines and HBEC30KT shown in (A) with Propidium iodide (PI) and Hoechst 33342 dyes. PI stained nuclei of all the cancer cell lines at 20 μM of PPS1D1, but not HBEC30KT cells. A known cell membrane damaging agent, BAC treatment caused PI stain on all the cells lines tested.

Previously, we found that PPS1D1 showed cytotoxic activity against HCC4017 lung cancer cells but not normal HBEC epithelial cells [5]. In this current study, we found that the target of PPS1D1 is PS and HCC4017 strongly expresses PS. Our next goal was to evaluate the level of PS externalization on other lung cancer cell lines and the activity profile of PPS1D1 on those cell lines. We treated a variety of lung cancer cell lines HCC4017 (lung adenocarcinoma), H460 (large cell lung cancer), H1395 (lung adenocarcinoma), HCC95 (squamous cell lung carcinoma), H1993 (adenocarcinoma; non-small cell lung cancer), H1695 and HBEC30KT (normal bronchial epithelial cells) with fluorescein-labeled Annexin V (FITC-Annexin V) and the bound fluorescein signal was detected using flow cytometry. As shown in Figure 5A, these cancer cell lines exhibited PS externalization with H460 and H1693 displaying the highest level (~ 65–70 % cells PS positive) while HBEC30KT normal cells with minimum levels. Next we performed standard cell viability (MTS) assay by treating these cell lines with PPS1D1 (1 and 20 μM) and 20 μM of a control compound PC462D1 (Supplementary Figure 6) in 96-well plates. PPS1D1 exhibited a strong cytotoxicity on all the cancer cell lines at 20 μM concentration but not at 1 μM (Figure 5B). This activity was somewhat similar when tested at 6, 12 and 24 hours post treatment using HCC4017 cancer cell line (Supplementary Figure 14). PPS1D1 had no effect on normal HBEC30KT cells at either concentration. These data are consistent with our previous observations that PPS1D1 has an IC_50_ ~ 10 μM on HCC4017 [5].

At this point we were interested in evaluating how PPS1D1 affects viability of these PS expressing cancer cell lines. While we understand the difficulties of narrowing down the exact mechanism of action of a compound targeting cell membrane lipids, we wanted to first evaluate whether PPS1D1 has any effect on cell membrane integrity. We treated cells with standard DNA staining dyes Propidium iodide (PI) and Hoechst 33342. While both bind DNA, only Hoechst is cell permeable. Therefore, when treating live cells only Hoechst will stain the nucleus while PI will stain the nucleus only if the cell membrane integrity is compromised. We treated all the lung cancer cell lines and normal HBEC30KT cells with these dyes in the presence of PPS1D1 (at 1 μM and 20 μM) or a control compound PC462D1 at 20 μM and evaluated fluorescence by microscopy without fixation. As shown in Figure 5C, untreated cells were only stained by the Hoechst dye. The control peptoid and PPS1D1 at 1 μM also only show staining with Hoechst dye. In contrast, treatment of lung cancer cells with PPS1D1 at 20 μM resulted in staining of cells with PI demonstrating a loss of membrane integrity. This effect was not observed on normal HBEC30KT cells. We also treated cells with the known cell membrane damaging agent Benzalkonium chloride (BAC), which resulted in PI-positive nuclei as shown in Figure 5C. These observations indicate that 20 μM PPS1D1 is selectively cytotoxic to PS-positive lung cancer cells.

**Figure 6:**
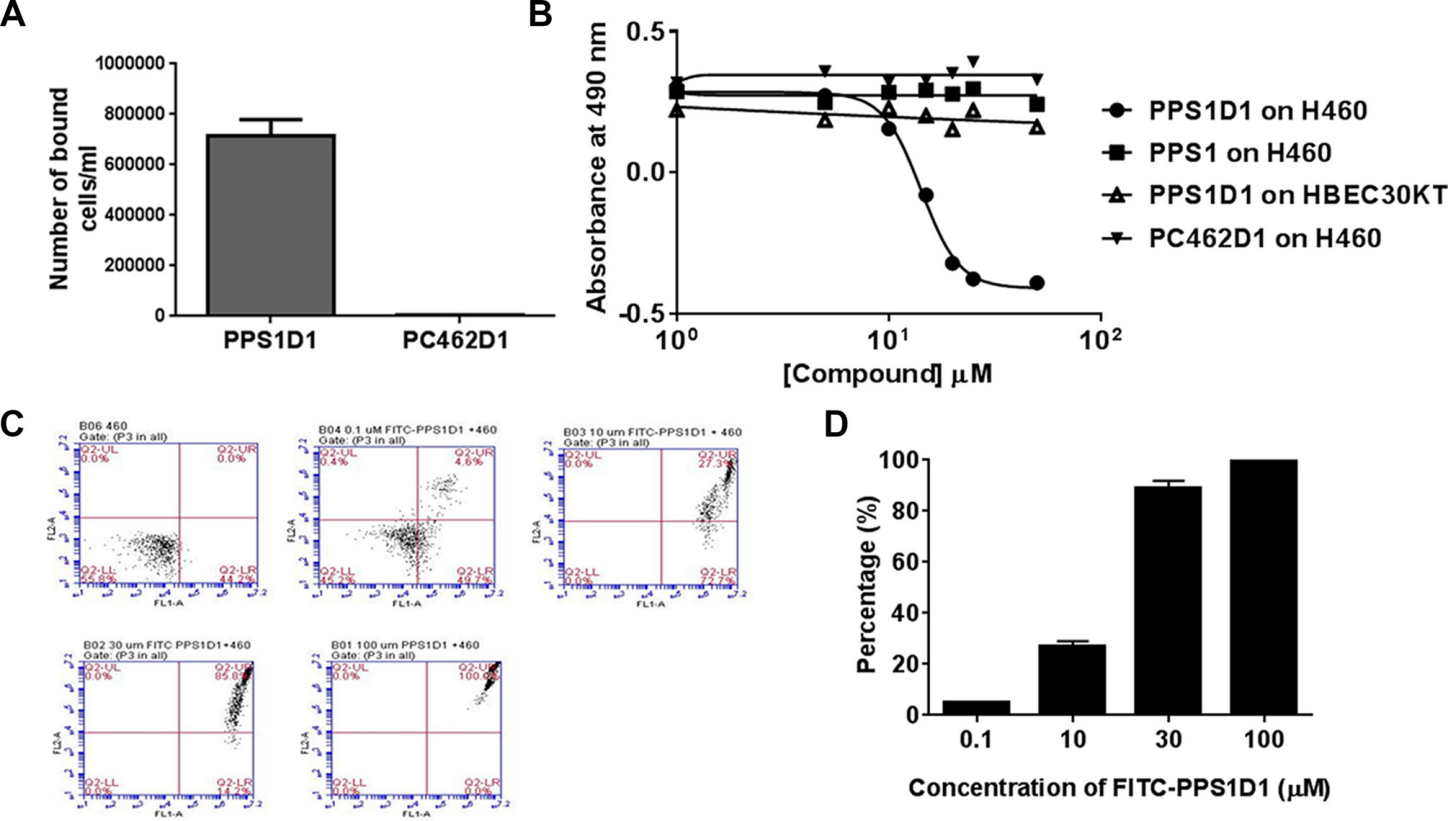
Comprehensive *in vitro* activity validation of PPS1D1 on H460 lung cancer cell line (**A**) Magnetic bead pulls down of H460 with PPS1D1, but not with control compound PC462D1 (Error bars represent standard deviation). (**B**) Standard MTS cell viability assay of H460 and normal HBEC30KT cells treated with PPS1D1, PPS1 and PC462D1. Only PPS1D1 induce the cell cytotoxicity on HCC4017, while no effect on normal HBEC30KT cells. (**C**) Flow cytometry studies of PPS1D1-FITC binding to H460 cells in the presence of Propidium iodide (PI). H460 cell population significantly moved to double positive region when PPS1D1-FITC concentration increases. (**D**) Quantification of FITC and PI double stained region.

We further tested the effect of PPS1D1 on H460 lung cancer cells in detail using several *in vitro* assays. H460 is an aggressive lung cancer cell line harboring mutations in p53 and KRAS that has been used widely in xenograft studies. PS expression is elevated on H460 cells (Figure 5A) and PPS1D1 shows cytotoxicity towards these cells (Figure 5B and 5C). We conducted a magnetic bead pulldown assay (Supplementary Experimental Procedure 9) by incubating 1 × 10^6^ H460 cells with magnetic bead coated PPS1D1 and control PC462D1 separately. We found that PPS1D1 coated magnetic beads readily retrieved about 75% of H460 cells compared to negligible amount pulled down by control compound PC462D1 (Figure 6A). Standard cell viability (MTS) assays (Supplementary Experimental Procedure 10) on H460 cells performed by treating increasing concentrations of PPS1D1, PPS1 and control compound PC462D1 in 96-well plates. PPS1D1 displayed very similar cytotoxic activity on H460 cells (IC_50_ = ~10 μM) as was observed for HCC4017 cells [5], while monomeric PPS1 and control PC462D1 did not affect H460 cells (Figure 6B). We again confirmed that PPS1D1 has no cytotoxicity on normal HBEC30KT cells (Figure 6B). Next we examined how the efficiency of PPS1D1 cytotoxicity increased with respect to the binding on H460 cancer cells at different concentrations using flow cytometry. PPS1D1-FITC was incubated for 1 hr with H460 cells at 0.1, 10, 30 and 100 μM concentrations and PI was added to stain dead cells. With increasing concentration of PPS1D1, the number of H460 cells double positive for PPS1D1-FITC and PI increased from ~5% to 100% (Figure 6C and 6D). These results indicate that PPS1D1 binds and is cytotoxic to H460 cells.

### Inhibition of growth of H460 lung cancer xenograft by PPS1D1

**Figure 7:**
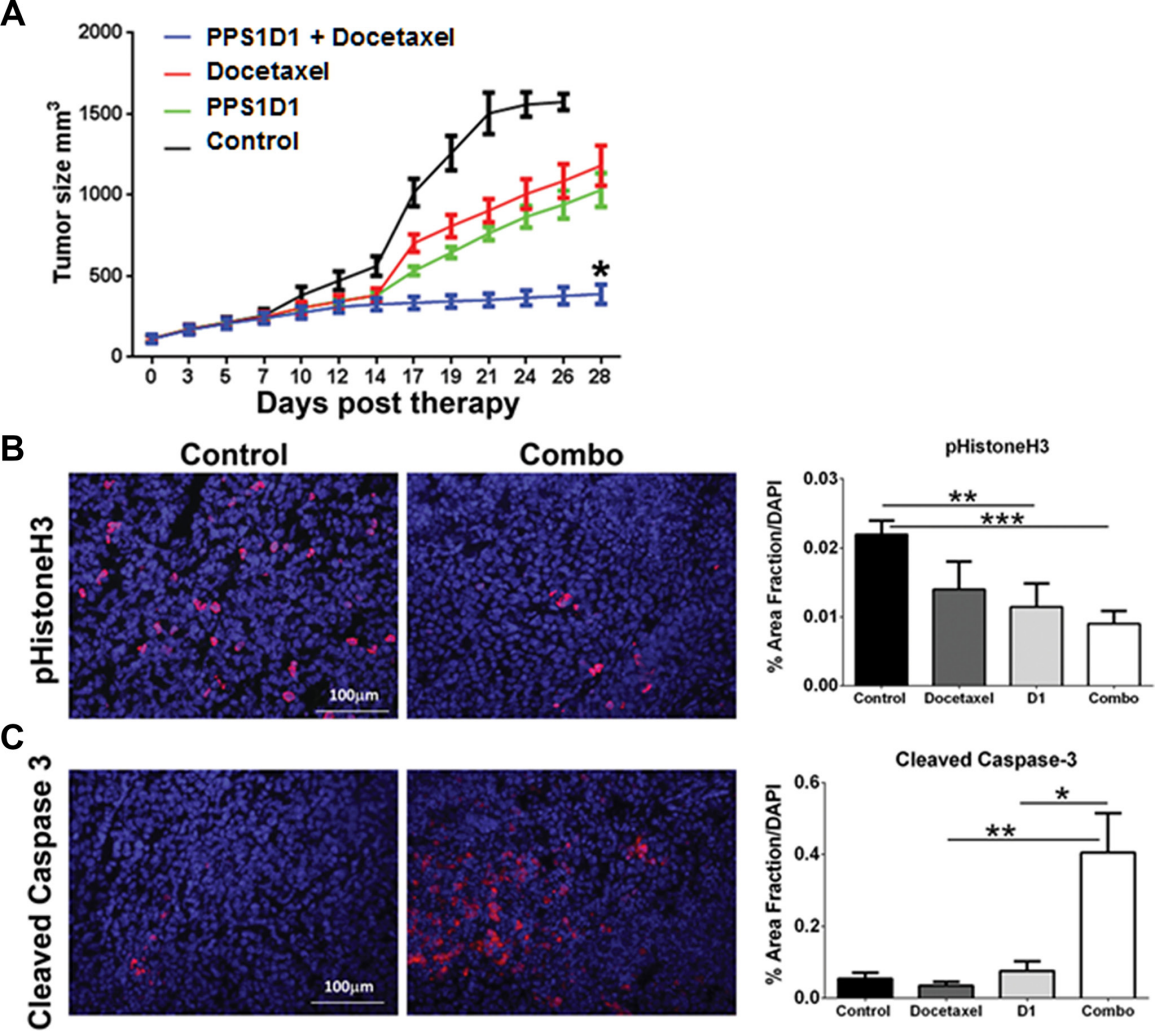
*In vivo* treatment of PPS1D1 on mice bearing H460 xenografts suppresses tumor growth (**A**) Mice bearing subcutaneous H460 xenografts were treated with PPS1D1 (D1, *n* = 8, 0.25 mg/mouse, 3 times per week on a M-W-F schedule), PC462D1 (Control, *n* = 8, 0.25 mg/mouse, 3 times per week on a M-W-F schedule), docetaxel (*n* = 8, 5 mg/kg, 2×/week), or the combination of PPS1D1 and docetaxel (n=8, combo). Mean +/− SEM tumor volume is displayed. PPS1D1 displayed tumor burden effects as a single agent as well as in combination with docetaxel. (**B, C**) Tumor tissue harvested after 4 weeks of therapy was evaluated for cell proliferation (B, phopsho-histone H3) and apoptosis (C, cleaved caspase-3) by immunofluorescence. DAPI was used as a counterstain and to normalize quantification of reactivity. *p < 0.05; **p < 0.01; ***p < 0.005. The PPS1D1 and docetaxel combination therapy strongly reduce cell proliferation and induce apoptosis.

We examined the effect of PPS1D1 on the growth of H460 xenografts implanted in NOD/SCID immunodeficient mice. Immunodeficient animals were chosen to facilitate the growth of human tumor xenografts. Although previous studies with PS-targeting bavituximab showed anti-tumor response required immune activation against tumor cells, our *in vitro* data suggests that PPS1D1 has a direct cytotoxic effect on cancer cells. Therapy with a control peptoid (PC462D1, 0.25 mg/mouse, 3×/week ip), PPS1D1 (0.25 mg/mouse, 3×/week ip), docetaxel (0.5 mg/kg, 2×/week, ip) or the combination of PPS1D1 + docetaxel was initiated when tumors were ~100 mm^3^ in volume. Figure 7A shows tumor volume vs days post therapy initiation and demonstrates that PPS1D1 and docetaxel slowed tumor growth as single agents. However, combination therapy was more effective than either therapy alone (Figure 7A). PPS1D1 at the doses used did not induce animal weight loss nor did it exacerbate toxicity induced by docetaxel (data not shown). Tumor tissue was harvested after 4 weeks of therapy and assessed for cell proliferation and apoptosis by immunohistochemistry. Combination therapy significantly reduced cell proliferation as measured by phosphorylated Histone H3 reactivity (Figure 7B) and significantly elevated apoptosis as determined by cleaved caspase 3 reactivity (Figure 7C). These data are consistent with the effect of other PS targeting agents (e.g., bavituximab), which showed enhanced activity in combination with standard therapy [26, 53]. It is clear from studies with bavituximab that standard chemotherapy (e.g., taxanes) increases the exposure of PS resulting in elevated binding of the PS targeting agent. We propose a similar mechanism underlies the enhanced activity of docetaxel and PPS1D1.

In conclusion, we showed that the anionic lipid-phosphatidylserine (PS) is the target of the anti-cancer peptide-peptoid hybrid PPS1, which was initially selected through a unique unbiased selection approach (OBTC cell screen) that compared HCC4017 lung cancer cells and HBEC30KT normal cells derived from the same patient [5]. This OBTC screening has many advantages for identification of targeting ligands specific for a selected cell population. The assay is dynamic with competing cell populations having equal access to targeting ligands. The screen is simple, rapid and economical and has been successfully employed to identify peptoids that bind therapeutically tractable targets previously [43, 54]. In the present study, we found that PPS1 preferentially binds to anionic phospholipids, most specifically to PS and then to PA, PI and PG to some extent, but not to PC, SM and PE. PS is exposed on tumor cells and endothelial cells in the tumor microenvironment of wide-varieties of tumor types [10, 55]. We found that a dimeric form of PPS1, PPS1D1, showed potent cytotoxicity towards series of lung cancer cells lines that express PS. PPS1D1 displayed potent cytotoxicity on H460 lung cancer cells *in vitro* and displayed single agent activity in H460 xenografts. PPS1D1 also potently enhanced the anti-tumor activity of docetaxel. There is an increasing need for anti-cancer agents that are effective against broad types of cancers, as the efficacy of protein targeted drugs are limited to certain subpopulations of cancer types due to the heterogeneous expressions of those protein drug targets. Phospholipid asymmetry and elevated PS levels is observed in the tumor microenvironments of most cancers analyzed to date. We propose that PPS1D1 may have efficacy in multiple tumor types and also has the potential to safely increase the efficacy of standard cancer therapy.

## MATERIALS AND METHODS

### Synthesis of PPS1

Synthesis of PPS1 compound was done on NovaSyn TGR resin (EMD Millipore, MA). First three amino acids, Fmoc-Met-OH, Fmoc–D-Lys(Boc)-OH and Fmoc-Lys(Boc)-OH were loaded to the resin after Fmoc removal each time. Then 5-mer peptoid region containing Boc-Diaminobutane, 4-methoxybenzylamine, (R)-Methylbenzylamine, Piperonylamine and (R)-Methylbenzylamine was completed using microwave assisted peptoid synthesis protocol. At the end, beads were washed with DCM and cleaved off with TFA cleavage cocktail (Please refer to Supplementary Experimental Procedures for detailed procedures).

### Synthesis of PPS1D1

PPS1D1 was synthesized on NovaSyn TGR resin. First, Fmoc-Lys(Fmoc)-OH was coupled overnight as the central linker, and both Fmoc groups were removed simultaneously allowing two copies of the sequence to be built on two amine groups of this central Lys. Beyond this point PPS1 synthesis procedure described above was utilized.

### Cell lines

HCC4017, H460, HCC95, H1693, H1395, HBEC30KT and HBEC3KT cell lines were obtained from the cell collection of Dr. John Minna's research group at UT-Southwestern Medical Center. HCC4017, H460, HCC95, H1693, and H1395 was grown in RPMI supplemented with 5% FBS. Normal lung cell line HBEC30KT and HBEC3KT were grown with keratinocyte serum free media (KSFM) supplemented with human recombinant epidermal growth factor and bovine pituitary extract.

### Cell staining

20,000 HCC4017 and HBEC30KT cells were plated in 8-well glass chamber plate. Incubation with control IgG or bavituximab (2 μg/ml, provided by Peregrine Pharmaceuticals, CA) was initiated 24 hrs post plating. The primary antibody was incubated for 1 hr 37ºC. Slides were washed in PBS two times and fixed with warm 4% paraformaldehyde (PFA) for 5-10 min at room temperature (RT) followed by washing 3 times with PBS. PFA was quenched with 50 mM NH_4_Cl (in PBS) for 5 min and washed 3 times with PBS. Reactivity was detected with goat-anti human Cy2 secondary antibody (1:1000) for 1hr at 37ºC. To visualize the cytoskeleton cells were permeabilized with PBS + 0.5% Triton-X100 for 5 min at room temperature, washed 2 times with PBS and stained with Texas Red conjugated phalloidin (1:200) for 20min. Slides were then mounted with Prolong Gold with DAPI (Invitrogen), cover slipped and evaluated by Olympus BX43 fluorescence microscope.

### Lipid ELISA-like binding assay

Lipids (Avanti Polar Lipids) were dissolved in hexane at 10 μg/ml and coated on to Immulon 1B “U” bottom microtiter plates (ThermoFisher, MA). Hexane was evaporated at room temp (in the hood) and the plates were blocked with 200 μl of 1% BSA in PBS for 1 hour. Plates were washed with 3× PBS. Serial dilutions (500 nM to 3.9 nM for PS vs PC assay – Figure 3A and 275 nM to 2.2 nM for other lipid binding assay – Figure 4A) of biotinylated PPS1D1 was dissolved in blocking buffer and added to wells (100 μl/well) and incubated for 1 hr on shaker. Plates were washed with 5× PBS and binding was detected with streptavidin-HRP (1:2000 in blocking buffer) followed by 100 μl OPD (Sigma-Aldrich). The reaction was stopped with 100 μl 0.18M H_2_SO_4_ and absorbance was read at 490 nm using the spectrophotometer (Spectramax i3, Molecular Devices, CA).

### Annexin V competition on lipid ELISA

Lipids (Avanti Polar Lipids) were dissolved in chloroform at 10 μg/ml and coated on to Immulon 1B “U” bottom microtiter plates (ThermoFisher, MA). Chloroform was evaporated at room temp (in the hood) and plates were blocked with 200 μl of 1% BSA for 1hour. Plates were washed with 3× PBS. Biotinylated PPS1D1 dissolved in blocking buffer was added and incubated for 1 hour at room temp. Plates were washed with 3× PBS and 2× Annexin binding buffer. 100nM Annexin V was added to the wells and incubated for 20 mins. Plates were washed with 3× PBS. Binding was detected with streptavidin-HRP (1:1000 in blocking buffer) followed by 100 μl OPD (Sigma-Aldrich, MO). The reaction was stopped with 100 μl 0.18M H2SO4 and absorbance was read at 490 nm using the spectrophotometer (Spectramax i3, Molecular Devices, CA).

### Liposome binding assay

Liposome binding assays were performed using two different types of liposomes, one containing 100 mol% 1-palmitoyl-2-oleoyl-sn-glycero-3-phosphocholine (POPC) and, another containing 85 mol%–15 mol% 1, 2-dioleoyl-sn-glycero-3-phospho-L-serine (DOPS). (Please refer to Supplementary Experimental Procedures for detailed procedures).

### Membrane lipid array

Membrane lipid arrays were purchased from Echelon Biosciences, UT. Membranes were blocked in 3% BSA in TBST for 1 hour and then incubated with 2.5 μg/ml of biotin-PPS1D1 for 2 hours. Membranes were washed with TBST and incubated with Streptavidin-HRP antibody at 1:750 dilutions (BioLegend, CA). After washing, signal was detected with ECL western blotting substrate (Life technologies, CA) using Fluorchem 8900 (Alpha Innotech Imaging system).

### Cell staining with Annexin V for PS expression

HCC4017, H460, HCC95, H1693, H1395 and HBEC30KT cells were dissociated from tissue culture plates with enzyme free cell dissociation buffer (Life technologies, CA). ~0.1 × 10^6^ cells were suspended in binding buffer (0.01 M HEPES/NaOH (pH 7.4), 0.14 M NaCl, and 2.5 mM CaCl_2_) and were treated with FITC-Annexin V and PI. After 15 minute incubation at RT, cells were analyzed by BD Accuri™ C6 flow cytometer.

### Cell viability assay

HCC4017, H460, HCC95, H1693, H1395 and HBEC30KT cells were grown in clear bottom 96 well plates. On second day, lung cancer cells were treated with PPS1, PPS1D1 and control PC462D1 in RPMI medium with 10% FBS containing 3% BSA. HBEC30KT was treated with PPS1, PPS1D1 and control PC462D1 in KSFM media with 3% BSA. On day 3, 20 μl of CellTiter 96^®^ AQueous One Solution (Promega, WI) was added to each well and absorbance was measured at 490 nm.

### Cell staining with Hoechst 33342 and Propidium iodide

10,000 cells of HCC4017, H460, HCC95, H1693, H1395 and HBEC30KT were plated on chamber slides (Lab-Tek, Thermo Fisher, MA). On second day, cells were treated with PPS1D1 (1μM and 20 μM), control PC462D1 (20 μM) and 0.005% Benzalkonium chloride (BAC) in RPMI medium with 10% FBS containing 3% BSA (KSFM media with 3% BSA for HBEC30KT). Chamber slides were washed with PBS three times. Cells were then stained with Hoechst 33342 (10 μg/ml) for 30 min in dark. Chamber slides were washed with 3× PBS. Cells were then stained with propidium iodide (1 mg/ml) for 15 mins. Cells were washed with 3× PBS and imaged using Fluorescence Microscope (Olympus BX-53).

### Magnetic bead binding assay

The assay was done using Dynabeads M-280 Streptavidin (Invitrogen). Nearly 9 × 10^6^ beads were transferred, re-suspended in PBS with 0.1% BSA. Biotinylated PPS1D1 and control PC462D1 were added to each vial and incubated for 30 minutes at RT. The beads were washed 3× PBS and 1 million H460 cells were added to each tube and incubated for 30 minutes at RT with gentle shaking. The bead bound cells were isolated by placing the vial on the magnet and after removing supernatant, cells were counted with hemocytometer.

### Animal studies

All animals were housed in a pathogen-free facility with continuous access to food and water. Experiments were approved by and performed in accordance with the Institutional Animal Care and Use Committee at the University of Texas Southwestern. Mice were purchased from the core breeding facility at UT Southwestern. Six- to eight-week-old female NOD/SCID mice were injected with 2.5 × 10^6^ H460 cells subcutaneously. Tumor volume was followed by twice weekly measurements with Vernier calipers. Animals were randomized and treatment was initiated with mean tumor volume of each group was 100 mm3. Four different groups used were: (I) control PC462D1, (II) PPS1D1, (III) docetaxel, and (IV) combination of PPS1D1 + docetaxel. Mice (*n* = 8/group) were treated with PPS1D1 or PC462D1 in saline by ip injection at a dose of 0.25 mg/mouse, 3 times per week on a M-W-F schedule. Docetaxel from the UT Southwestern Clinical pharmacy was diluted in saline and delivered 2×/week ip at 5 mg/kg. Animals were sacrificed after 4 weeks of therapy. Tumor tissue was snap frozen, sectioned and stained with antibodies specific for phospho- Histone H3 (Millipore, #06-570) and cleaved caspase 3 (Cell Signaling, #9664). Reactivity was developed with appropriate fluorescently conjugated secondary antibodies (Jackson ImmunoResearch) and mounted with Prolong Gold with DAPI (Invitrogen), coverslipped and evaluated by fluorescence microscopy.

## SUPPLEMENTARY MATERIALS FIGURES


