# Supplementary material for: Identification of lipid-phosphatidylserine (PS) as the target of unbiasedly selected cancer specific peptide-peptoid hybrid PPS1
Source: Oncotarget. 2016 Apr 22;7(21):30678–90. doi: 10.18632/oncotarget.8929 (PMC5058709; doi:10.18632/oncotarget.8929)
Supplement: Supplementary file 1 [file oncotarget-07-30678-s001.pdf]

Identification of lipid-phosphatidylserine (PS) as the target of unbiasedly selected cancer specific peptide-peptoid hybrid PPS1

Supplementary Materials

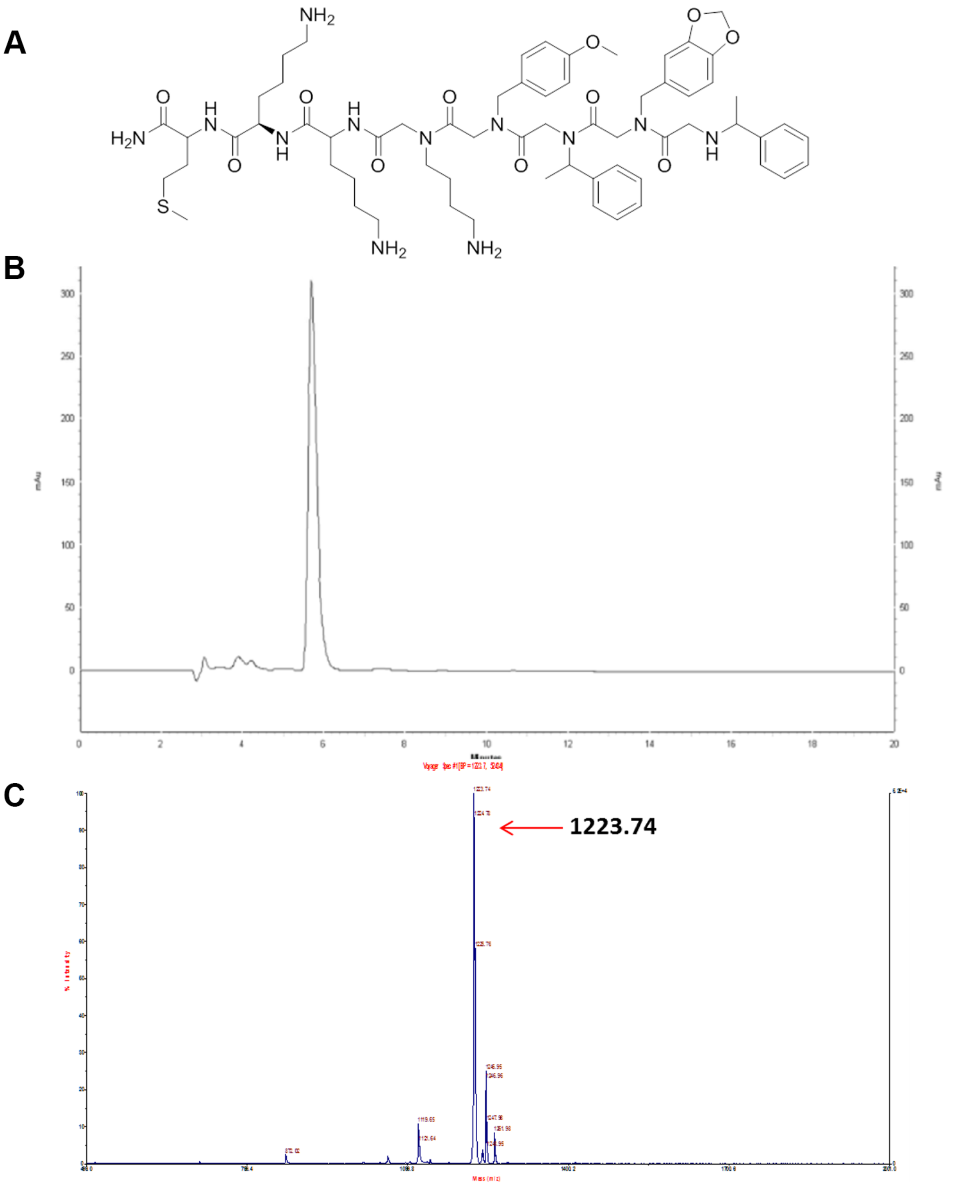

Supplementary Figure S1: Characterization of PPS1: (A) Chemical structure of PPS1, (B) Analytical HPLC of PPS1, (C) MALDI-TOF spectrum of PPS1.

A

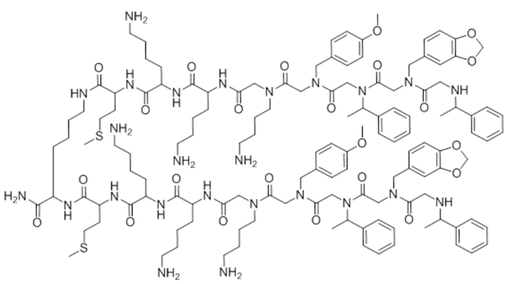

B

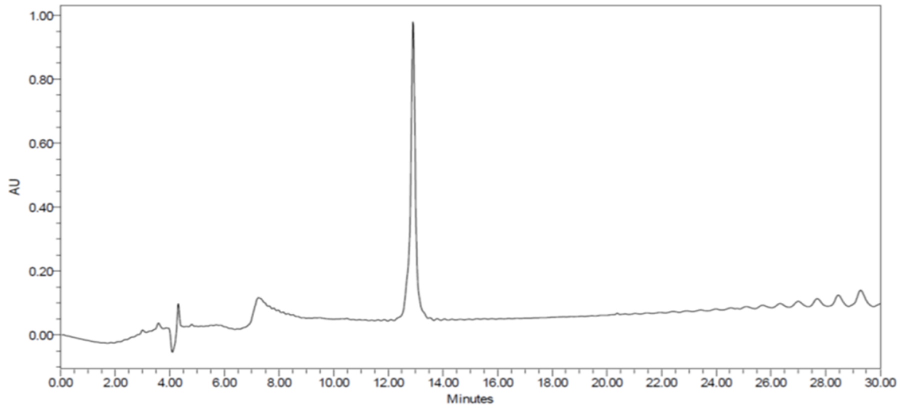

C

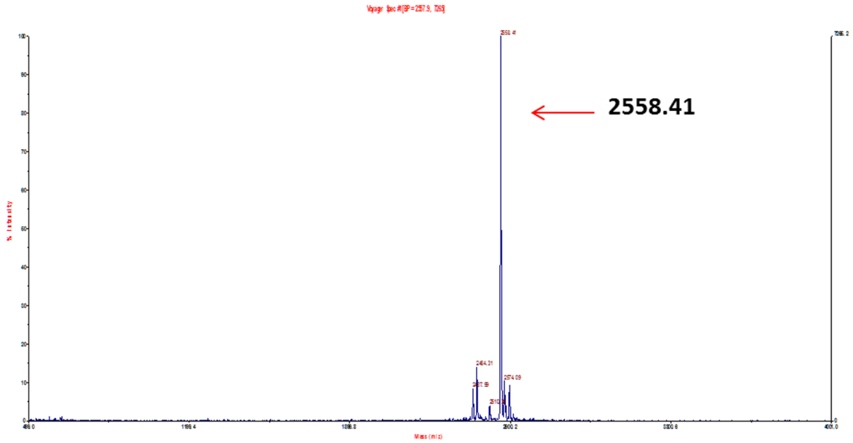

Supplementary Figure S2: Characterization of PPS1D1: (A) Chemical structure of PPS1D1, (B) Analytical HPLC of PPS1D1, (C) MALDI-TOF spectrum of PPS1D1.

A

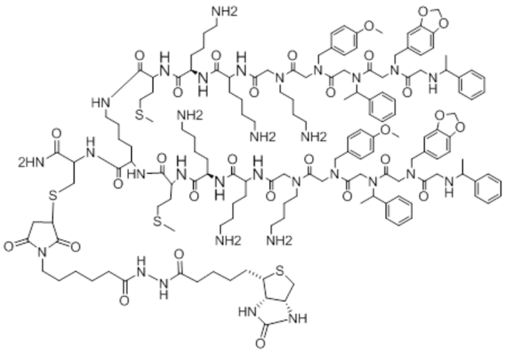

B

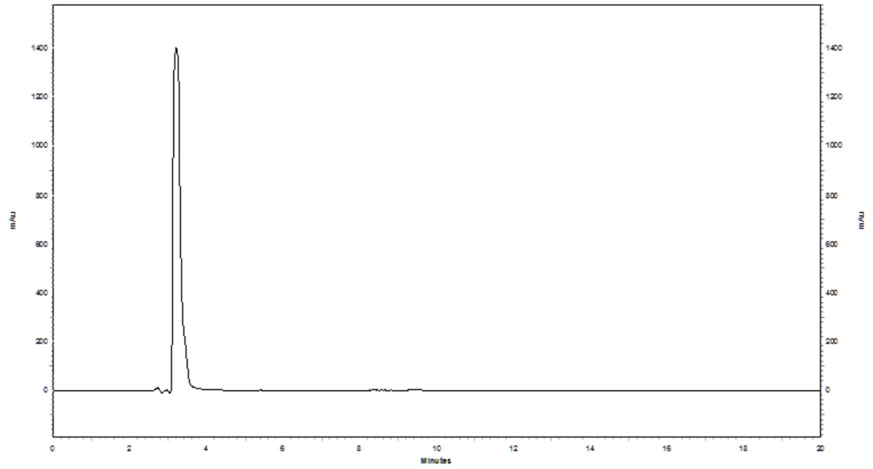

C

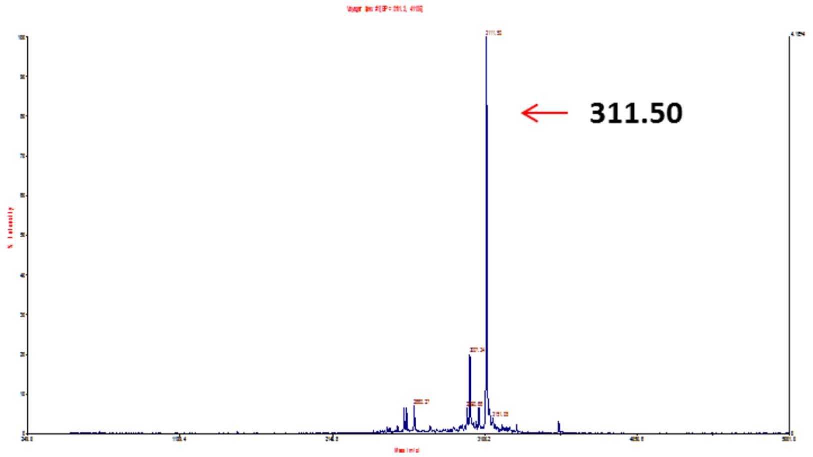

Supplementary Figure S3: Characterization of biotinylated PPS1D1: (A) Chemical structure of biotinylated PPS1D1, (B) Analytical HPLC of biotinylated PPS1D1, (C) MALDI-TOF spectrum of biotinylated PPS1D1.

A

B

C

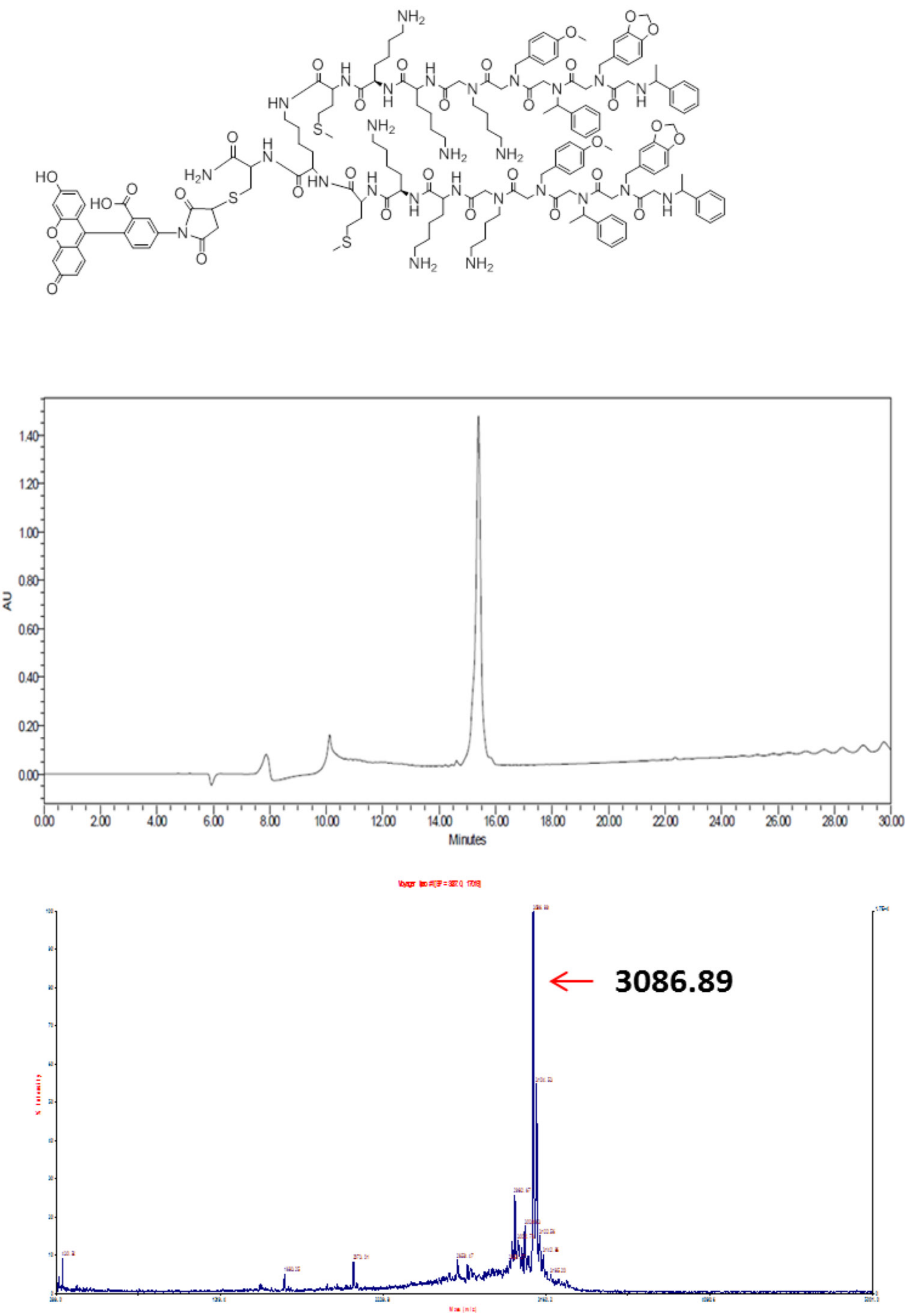

Supplementary Figure S4: **Characterization of FITC-PPS1D1: (A) Chemical structure of FITC-PPS1D1, (B) Analytical HPLC of FITC-PPS1D1, (C) MALDI-TOF spectrum of FITC-PPS1D1.**

A

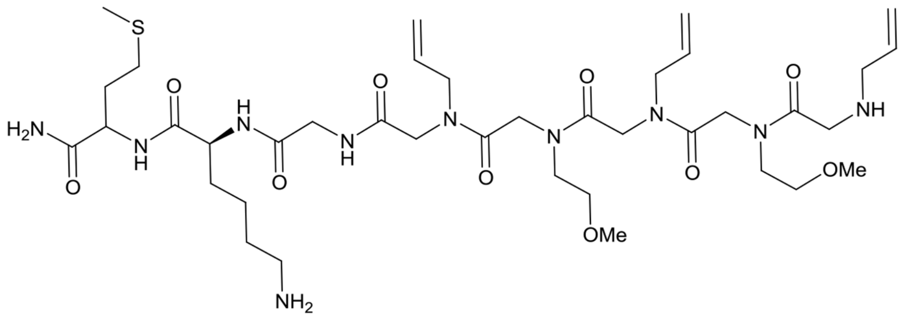

B

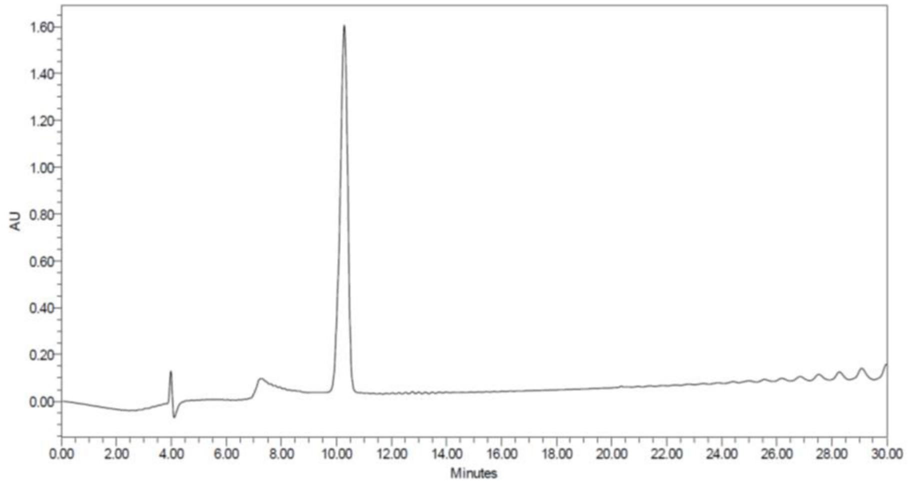

C

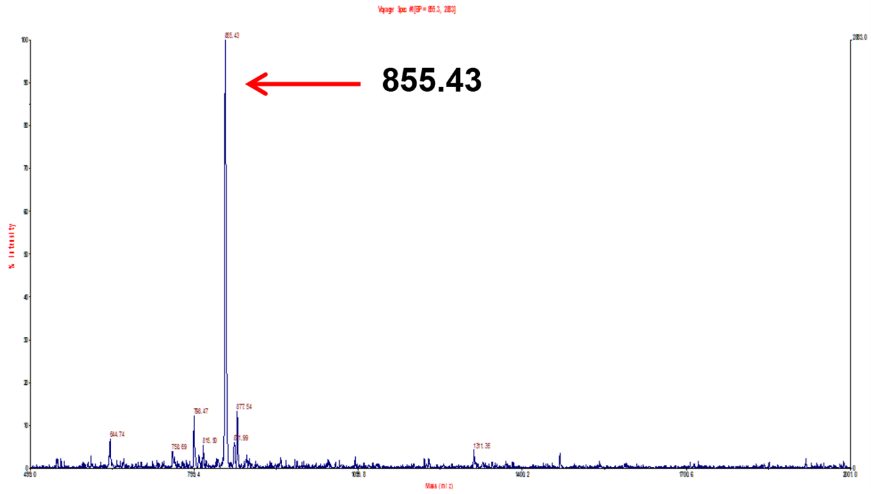

Supplementary Figure S5: Characterization of PC462: (A) Chemical structure of PC462, (B) Analytical HPLC of PC462, (C) MALDI-TOF spectrum of PC462.

A

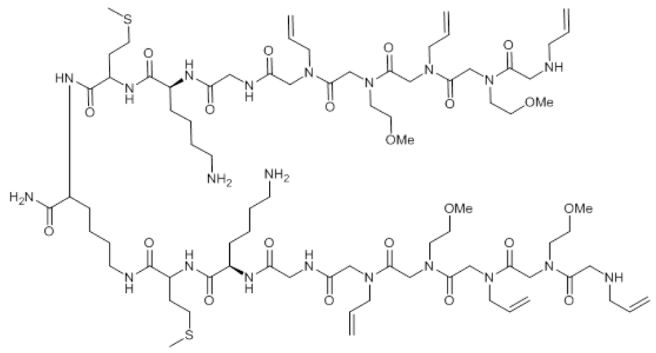

B

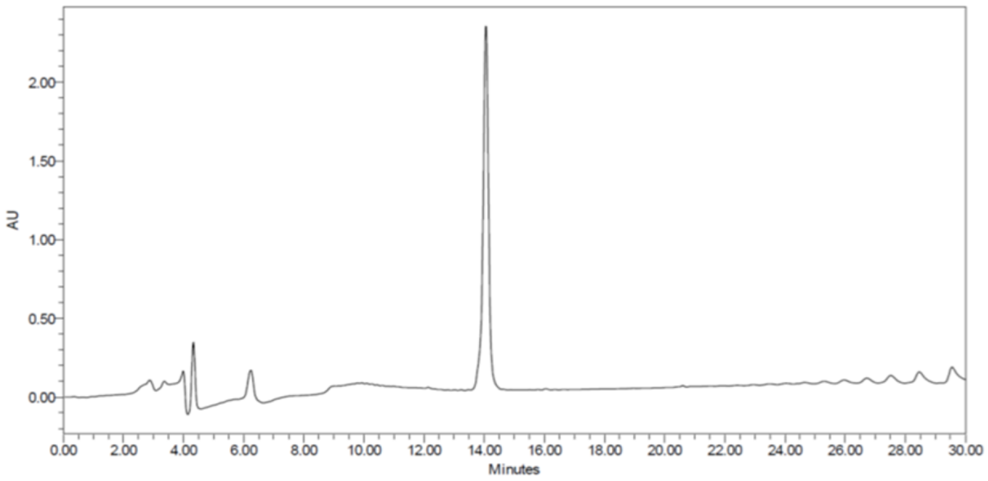

C

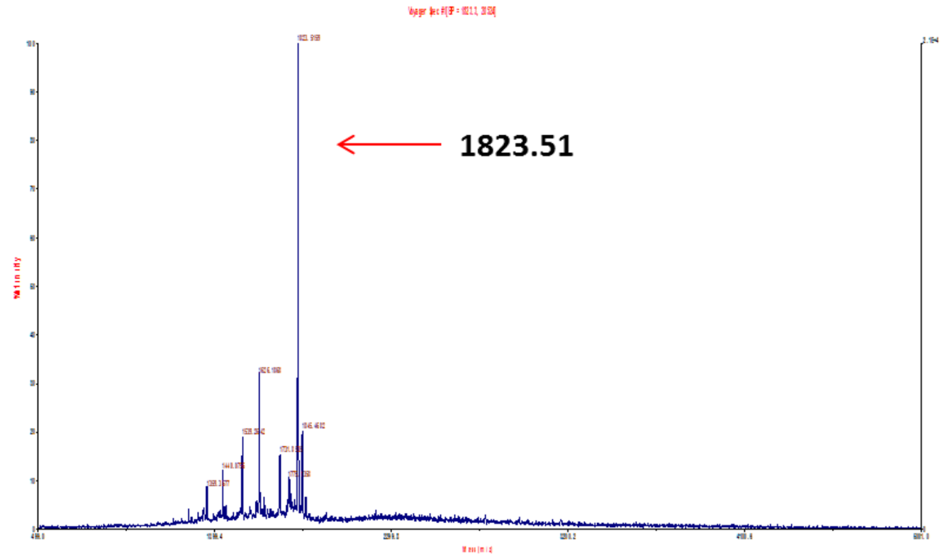

Supplementary Figure S6: Characterization of PC462D1: (A) Chemical structure of PC462D1, (B) Analytical HPLC of PC462D1, (C) MALDI-TOF spectrum of PC462D1.

A

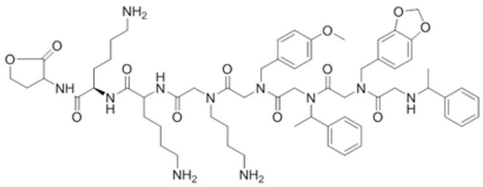

B

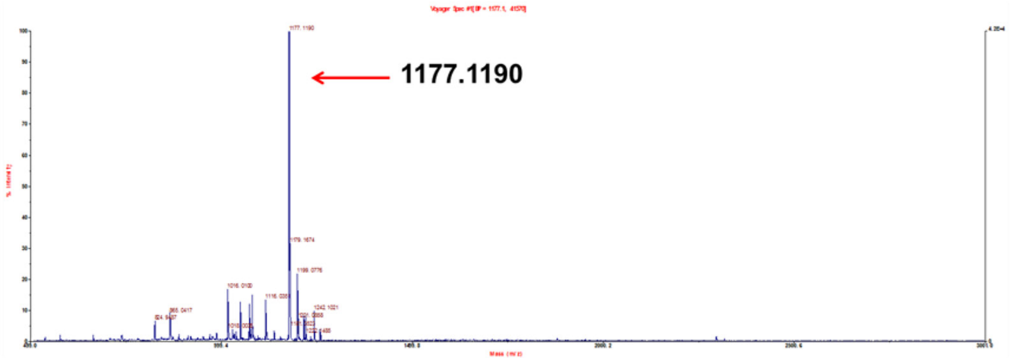

Supplementary Figure S7: **Characterization of PPS1: (A) Chemical structure of PPS1 (cleaved with cyanogen bromide) synthesized on Tentagel MB-NH2 beads, (B) MALDI-TOF spectrum of PPS1 after cleavage from Tentagel MB-NH2 beads.**

A

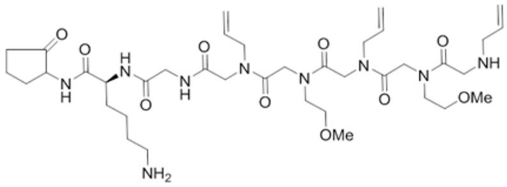

B

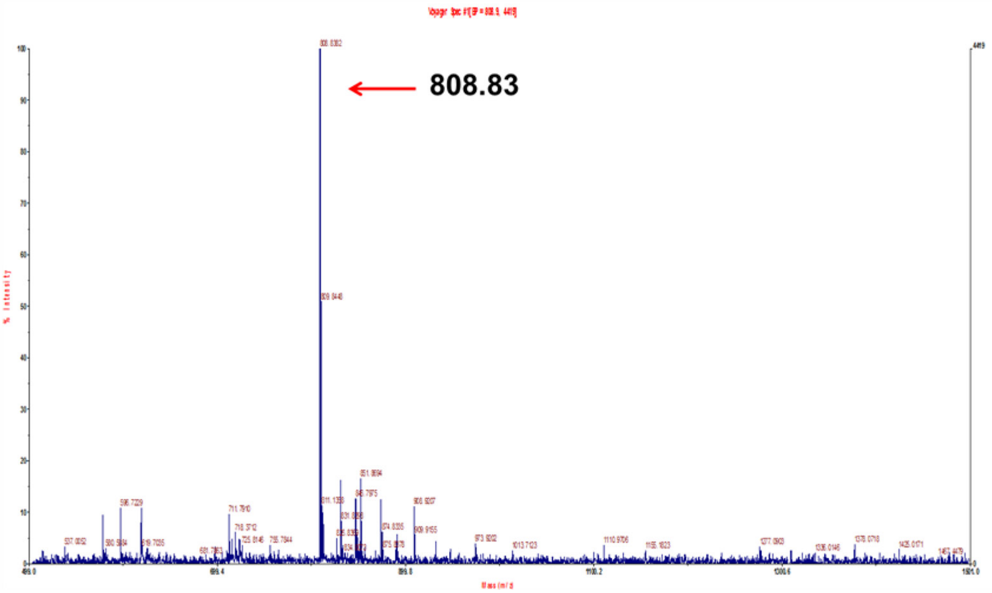

Supplementary Figure S8: **Characterization of PC462: (A) Chemical structure of PC462 (cleaved with cyanogen bromide) synthesized on Tentagel MB-NH2 beads, (B) MALDI-TOF spectrum of PC462 after cleavage from Tentagel MB-NH2 beads.**

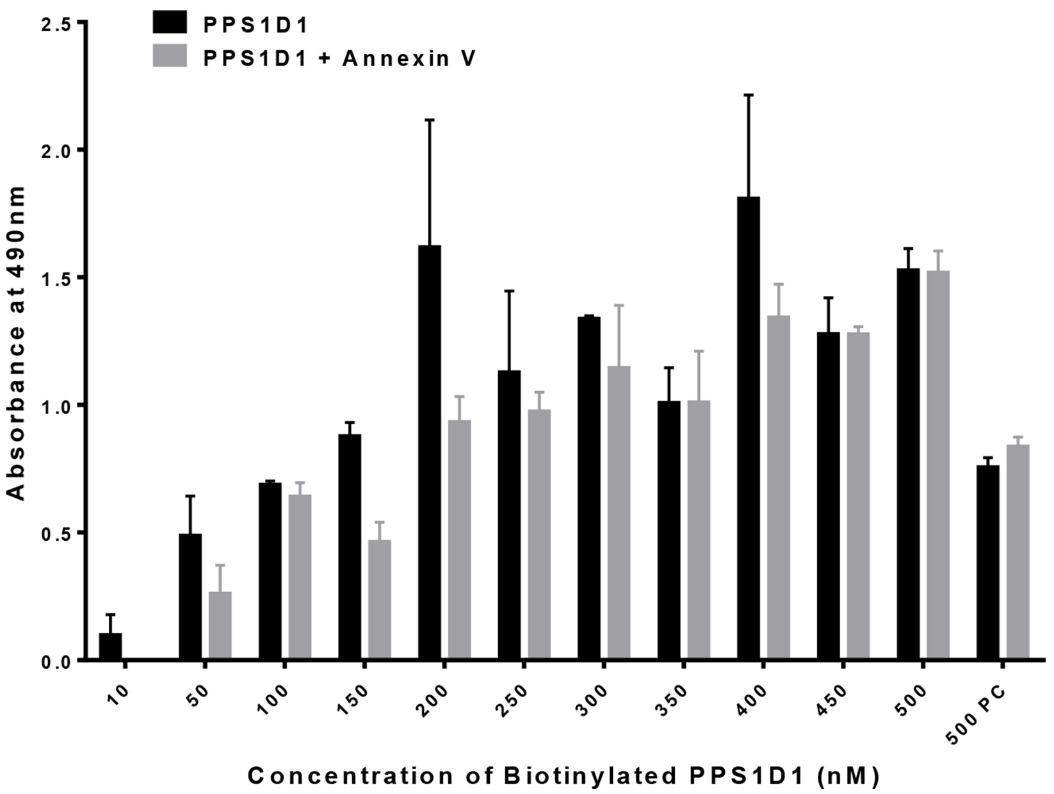

Supplementary Figure S9: Unlabeled Annexin V did not compete with FITC-PPS1D1 binding on an ELISA-like binding assay.

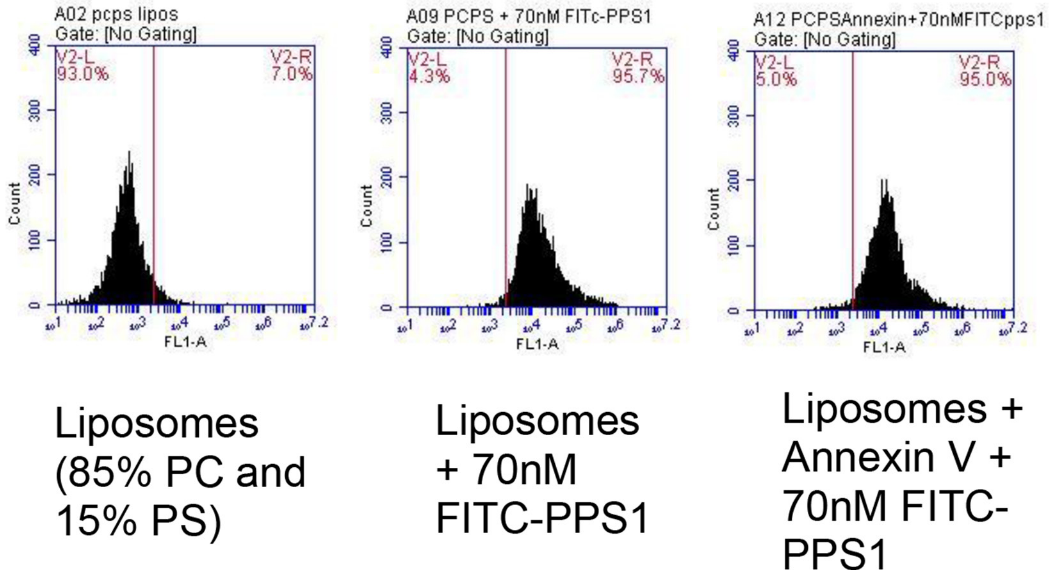

Supplementary Figure S10: Unlabeled Annexin V did not compete with FITC-PPS1 binding to liposomes made with 85% PC – 15% PS.

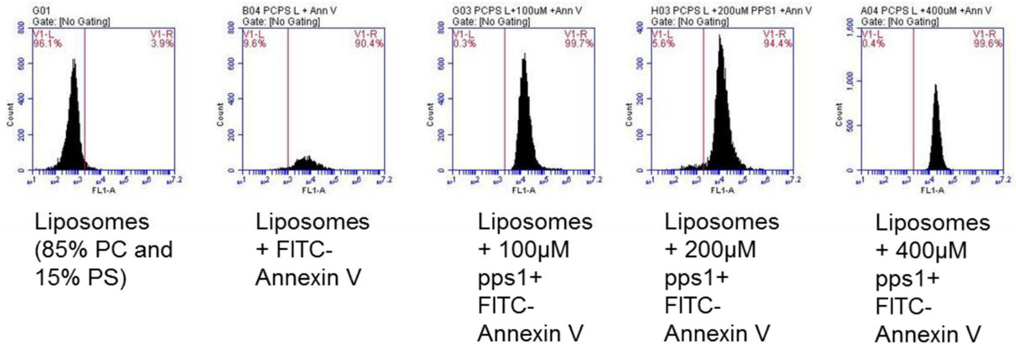

Supplementary Figure S11: Unlabeled PPS1 did not compete with FITC- Annexin V binding to liposomes made with 85% PC – 15% PS.

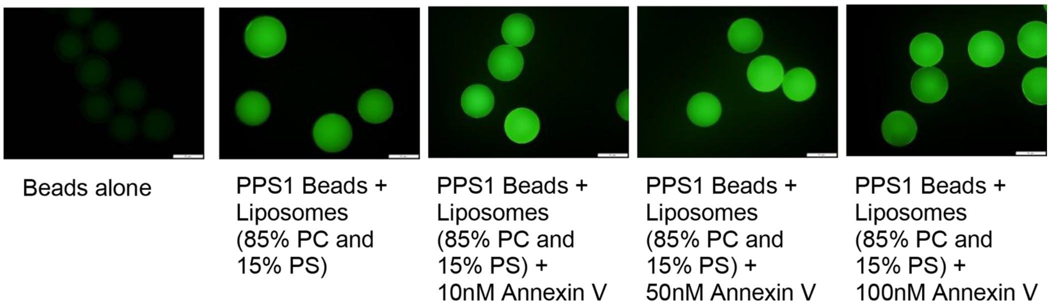

Supplementary Figure S12: Liposomes (85% PC – 15% PS) incorporated with fluorophore NBD and then competed with Annexin V at 10, 50 and 100 nM. None of these conditions were able to remove liposomes from beads.

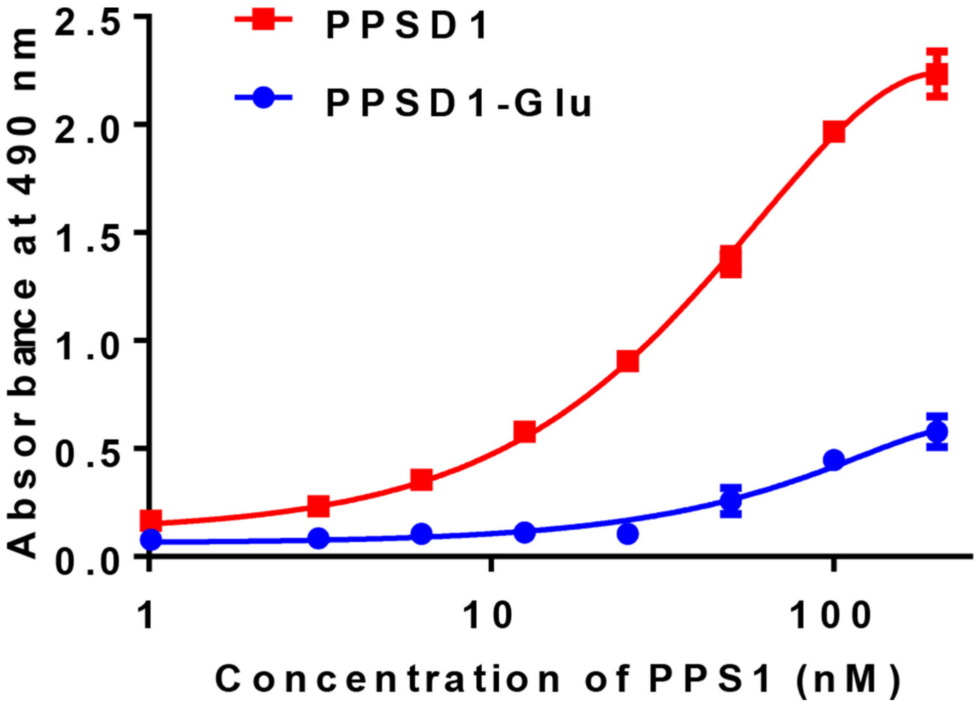

Supplementary Figure S13: ELISA binding assay of PPS1D1-FITC and PPS1D1-Glu-FITC [replacing one of the positively charged lysine residues (3rd residue from C-terminal) of PPS1D1] with phosphotidylserine (PS) indicates that PPS1D1-Glu-FITC loses its binding ability when positive charges are converted to negative charges.

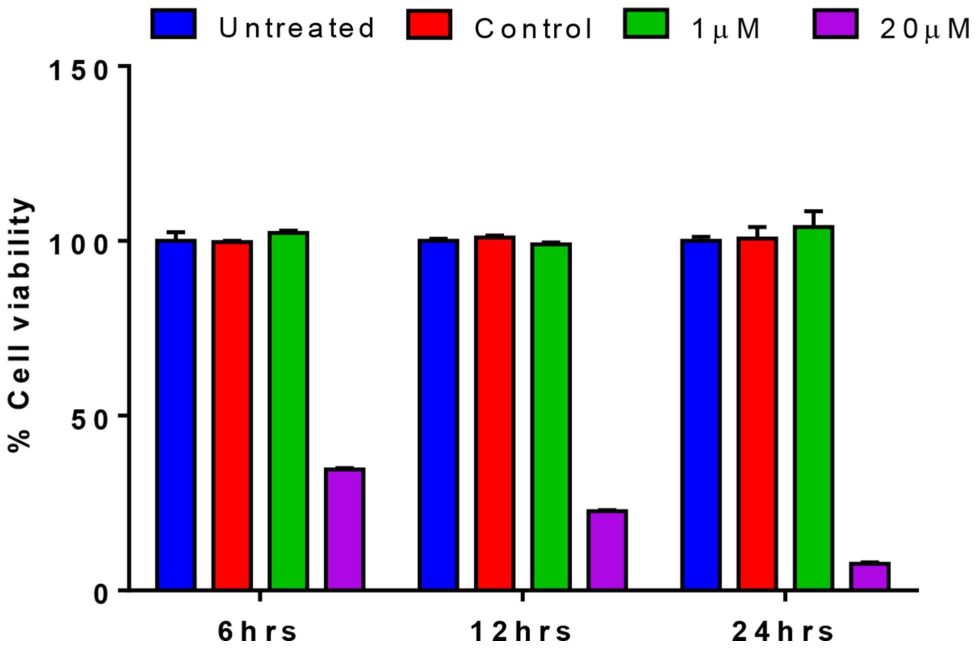

Supplementary Figure S14: MTS assay results of PPS1D1 and control PC462D1 on HCC4017 cell line evaluated at 6, 12 and 24 hours.

Supplementary experimental procedure 1: Synthesis of PPS1. NovaSyn TGR resin (EMD Millipore, MA) 150 mg were swelled in dimethylformamide (DMF, Acros Organics, NJ) for 30 min at room temperature in a 5 ml reaction vessel (intavis AG, Germany). The reaction vessels were drained and treated with 2 M Fmoc-Met-OH amino acid (with coupling agents HBTU, HOBt and DIPEA) in anhydrous DMF (Sigma-Aldrich, MO). Then the reaction vessel was placed on a shaker for overnight, drained and washed with DMF (5 ml × 10 times). Fmoc group was removed by treating the beads with 20% piperidine (Sigma-Aldrich, MO) for 10 minutes twice on the shaker. After washing the reaction vessel, subsequent amino acids Fmoc-D-Lys(Boc)-OH and Fmoc-Lys(Boc)-OH were added (for 2 h reaction time) with each time removing Fmoc group as described above. Then the 5-mer peptoid region was synthesized using microwave assisted peptoid synthesis protocol. Reaction vessels were treated with 2 M Bromoacetic acid in anhydrous DMF (1 ml) and 2M DIC in anhydrous DMF (1 ml), gently shaken for 30 seconds and microwaved (1000 W) for 15 seconds with the power set at 10%. The beads were shaken again for about 15 seconds and microwaved another round as described above. The reaction vessel was drained and washed with DMF (2 ml × 10 times). Then the reaction vessel was treated with 1 M solution of the primary amine (2 ml) and microwaved two times for 15 seconds after gentle shaking. The primary amines used were Boc-Diaminobutane, 4-methoxybenzylamine, (R)-Methylbenzylamine, Piperonylamine and (R)-Methylbenzylamine. At the end of synthesis the beads were washed with Dichloromethane (DCM) (2 ml × 10 times), and the compound was cleaved with 2.5 ml of cleavage cocktail containing 95% Triflouoroacetic acid (TFA), 2.5% water and 2.5% Triisopropylsialine(TIS) on the shaker for 2 hours and compound was purified using HPLC. Synthesis was confirmed using MALDI-TOF MS (Voyager DePro, AB Systems, MA).

Supplementary experimental procedure 2: Synthesis of PPS1D1. PPS1D1 was synthesized on NovaSyn TGR resin (EMD Millipore, MA). First, Fmoc-Lys(Fmoc)-OH was coupled overnight as the central linker, and both Fmoc groups were removed simultaneously allowing two copies of the sequence to be built on two amine groups of this central Lys. First three amino acids, Fmoc-Met-OH, Fmoc–D-Lys(Boc)-OH and Fmoc-Lys(Boc)-OH were loaded to the resin after Fmoc removal each time. Then 5-mer peptoid region containing Boc-Diaminobutane, 4-methoxybenzylamine, (R)-Methylbenzylamine, Piperonylamine and (R)-Methylbenzylamine was completed using microwave assisted peptoid synthesis protocol. At the end of synthesis the beads were washed with Dichloromethane (DCM) (2 ml × 10 times), and the compound was cleaved with 2.5 ml of cleavage cocktail containing 95% Triflouoroacetic acid (TFA), 2.5% water and 2.5% Triisopropylsialine(TIS) on the shaker for 2 hours and compound was purified using HPLC. Synthesis was confirmed using MALDI-TOF MS (Voyager DePro, AB Systems, MA).

Supplementary experimental procedure 3: Synthesis of biotinylated PPS1D1. This synthesis was carried out on NovaSyn TGR resin (EMD Millipore, MA). Fmoc-Cys(Trt)-OH (HOBt, HBTU,DIPEA) was loaded as first amino acid on to the resin and the rest of the PPS1D1 synthesis was conducted as described previously. At the end 95% TFA, 2.5% water and 2.5% TIS mixture was used to cleave the compound from resin and to remove the side chain protection. Then the TFA was evaporated and resulting solid compound was dissolved in 1:1 water: Acetonitrile (ACN) mixture. This solution was subjected to HPLC purification using the solvent conditions starting from 100:0 water: ACN to 50:50 water: ACN. The purified compound was lyophilized to obtain the dry product. Biotin-5–maleimide (Thermofisher,

MA) dissolved in DMSO was coupled to this compound (1 M: 1 M ratio) in buffer solution at pH 7. The coupled Biotinylated PPS1D1 compound was purified with HPLC. Synthesis was confirmed using MALDI-TOF MS (Voyager DePro, AB Systems, MA).

Supplementary experimental procedure 4: Synthesis of FITC-PPS1D1. This synthesis was carried out on NovaSyn TGR resin (EMD Millipore, MA). Fmoc-Cys(Trt)-OH (HOBt, HBTU,DIPEA) was loaded as first amino acid on to the resin and the rest of the PPS1D1 synthesis was conducted as described previously. At the end 95% TFA, 2.5% water and 2.5% TIS mixture was used to cleave the compound from resin and to remove the side chain protection. Then the TFA was evaporated and resulting solid compound was dissolved in 1:1 water: Acetonitrile (ACN) mixture. This solution was subjected to HPLC purification using the solvent conditions starting from 100:0 water: ACN to 50:50 water: ACN. The purified compound was lyophilized to obtain the dry product. Fluorescein-5–maleimide (Thermofisher,MA) dissolved in DMSO was coupled to this compound (1 M: 1 M ratio) in buffer solution at pH 7. The coupled FITC-PPS1D1 compound was purified with HPLC.

Supplementary experimental procedure 5: Synthesis of control compound PC462. PC462 was synthesized on NovaSyn TGR resin (EMD Millipore, MA).First amino acid Fmoc-Met-OH was coupled overnight, the next two amino acids Fmoc–D-Lys(Boc)-OH and Fmoc-Gly-OH were loaded to the resin with removing Fmoc group each time. Then the 5-mer peptoid region containing Allyamine and 2-Methoxyethylamine was synthesized using microwave assisted peptoid synthesis protocol as described previously. At the end of synthesis the beads were washed with Dichloromethane (DCM) (2 ml × 10 times), and peptoid was cleaved with 2.5 ml of cleavage cocktail containing 95% Triflouoroacetic acid (TFA), 2.5% water and 2.5% Triisopropylsialine(TIS) on the shaker for 2 hours and compound was purified using HPLC. Synthesis was confirmed using MALDI-TOF MS (Voyager DePro, AB Systems).

Supplementaryexperimental procedure 6: Synthesis of control compound PC462D1. PC462D1 was synthesized on NovaSyn TGR resin (EMD Millipore, MA).First, Fmoc-Lys(Fmoc)-OH was coupled overnight as the central linker, and both Fmoc groups were removed simultaneously allowing two copies of the sequence to be built on two amine groups of this central Lys. Then first three amino acids Fmoc-Met-OH, Fmoc–D-Lys(Boc)-OH and Fmoc-Gly-OH were loaded to the resin with removing Fmoc group each time. Then the 5-mer peptoid region containing Allyamine and 2-Methoxyethylamine was synthesized using microwave assisted peptoid synthesis protocol as described previously. At the end of synthesis the beads were washed with Dichloromethane (DCM) (2 ml × 10 times), and peptoid was cleaved with 2.5 ml of cleavage cocktail containing 95% Triflouoroacetic acid (TFA), 2.5% water and 2.5% Triisopropylsialine(TIS) on the shaker for 2 hours and compound was purified using HPLC. Synthesis was confirmed using MALDI-TOF MS (Voyager DePro, AB Systems).

Supplementary experimental procedure 7: Synthesis of PPS1 on Tentagel. This synthesis was carried out on Tentagel MB-NH2 beads(Rapp Polymere,Germany).First amino acid Fmoc-Met-OH was coupled overnight and the rest of the PPS1 synthesis was conducted as described previously. At the end cynogen bromide cleavage was performed. Small amounts of beads were removed from reaction vessels before storage and washed with DCM (2 ml × 3 times). 30 mg/ml CNBr solution (1 ml) was prepared in 5:4:1 Acetonitrile: Acetic acid: water. 50 µl from this solution was added to the beads and kept on the shaker overnight. CNBr solution was allowed to evaporate and 1:1 mixture of acetonitrile and water was added to the beads and resulting solution was used to confirm mass of the compound.

Supplementary experimental procedure 8: Synthesis of PC462 on Tentagel. This synthesis was carried out on Tentagel MB-NH2 beads(Rapp Polymere,Germany).First amino acid Fmoc-Met-OH was coupled overnight and the rest of the PC462 synthesis was conducted as described previously. At the end cynogen bromide cleavage was performed. Small amounts of beads were removed from reaction vessels before storage and washed with DCM (2 ml × 3 times). 30 mg/ml CNBr solution (1 ml) was prepared in 5:4:1 Acetonitrile: Acetic acid: water. 50 µl from this solution was added to the beads and kept on the shaker overnight. CNBr solution was allowed to evaporate and 1:1 mixture of acetonitrile and water was added to the beads and resulting solution was used to confirm mass of the compound.

Supplementary experimental procedure 9: Magnetic bead pull down assay with PPS1. This assay was performed with Dynabeads M-280 Streptavidin (Life technologies,CA). First the beads were re-suspended in the original vial by vortexing. From this 14 µl of beads (approximately 9×10^6 beads) were transferred to a microcentrifuge tube and 500 µl of PBS with 0.1% BSA was added. The microcentrifuge tube containing the beads was placed on the magnet for 2 minutes and the supernatant was removed by aspiration. The beads were washed three times with 500 µl of PBS with 0.1% BSA. Then biotinylated PPS1D1 or PC462 were added to each vial and the reaction was incubated for 30 minutes at room temperature with gentle shaking. Then the beads were washed 3 times with 500 µl of PBS with 0.1% BSA. H460 cells (1×10^6 cells in 1ml of RPMI with 1% BSA) were added to each vial and incubated for 30 minutes at room temperature with gentle shaking. The supernatant was removed and numbers of bead bound cells were calculated using hemocytometer.

Supplementary experimental procedure 10: MTS viability assay on H460 cells. 5,000 of H460 cells and HBEC30KT cells were grown in each well of a white clear bottom 96 well plates (Corning Inc, NY) on day 1 of the experiment. On day 2, four experimental sets were designed to treat the wells with graded concentrations of PPS1, PPS1D1 and control compound PC462D1 prepared in RPMI medium containing 5% FBS with 3% BSA for H460 cells treatment and PPS1D1 on Keratinocyte-SFM with 3% BSA media for HBEC30KT treatment. Eight graded concentrations ranging from 0.01 µM – 50 µM were used for all compounds and each concentration was done in triplicates. 6 wells were left untreated as controls. On day 4, media was removed from each well and treatment was repeated as described previously. On day 5, 20 µl of CellTiter 96® AQueous One Solution Cell Proliferation Assay (Promega, WI) was added to each well and absorbance was measured at 490 nm using a plate reader 2 hours after treatment.

Supplementary experimental procedure 11: Competition on liposomes.

Liposome preparation

1-palmitoyl-2-oleoyl-sn-glycero-3-phosphocholine (POPC) and 1, 2-dioleoyl-sn-glycero-3-phospho-L-serine (DOPS) were purchased from Avanti Polar Lipids (Alabaster, AL). POPC and DOPS were mixed in an 85:15 molar ratio for PS containing liposomes. For PC containing liposomes POPC alone was used. Dried lipids were re-suspended in Tris buffer solution (10 mM, pH 7.4) to 1 mg/ml. This stock was fully mixing and exposed to more than 5 freeze-thaw cycles. A mini-extruder from Avanti Polar Lipids and Whatman Nuclepore Track-Etch 1.0 µm filters from GE Healthcare were used to produce liposomes. To produce 7-nitro-2-1,3-benzoxadiazol-4-yl (NBD) labeled liposomes 1 mol% was added to lipid mixes.

Unlabeled Annexin V vs FITC-PPS1 competition

0.1 mM liposomes (85 mol% POPC, 15 mol% DOPS) were incubated with incubated with 50nM Annexin V in Binding Buffer (0.01 M HEPES/ NaOH (pH 7.4), 0.14 M NaCl, and 2.5 mM CaCl2) with 3% BSA for 1hour. FITC PPS1 was added and the solution was incubated for 1hour at room temperature. The binding of FITC-PPS1 to liposomes was analyzed by BD Accuri™ C6 flow cytometer (Becton-Dickinson, NJ). All data were analyzed using BDAccuriC6 software.

Unlabeled PPS1 vs FITC-Annexin V competition

0.1 mM liposomes (85 mol% POPC, 15 mol% DOPS) were incubated PPS1 in Binding buffer with 3% BSA for 1hour. 50 nM FITC Annexin V was added to the solution and the solution was incubated for 1 hour at room temperature. The binding of FITC-Annexin V to liposomes was analyzed by BD AccuriTM C6 flow cytometer (Becton-Dickinson, NJ). All data were analyzed using BDAccuriC6 software.

Supplementary experimental procedure 12: On-bead competition assay. PPS1 and PC462 were synthesized on Tentagel beads. Organic solvent was removed and the beads were washed with 3 times with PBS. The beads were then incubated with PBS containing 3% BSA for 1hour at room temperature. These beads were then incubated with 0.1 mM liposomes (84 mol% POPC, 15 mol% DOPS and 1 mol% NBD) for 2hours at room temperature. Beads containing liposomes were the washed with binding buffer 3 times. 50 nM Annexin V was added to the beads and incubated for 2 hours at room temperature. Beads were then washed with binding buffer and imaged using fluorescence Microscope (Olympus BX-53).
